# Gene network analysis for identification of microRNA biomarkers for asthma

**DOI:** 10.1186/s12931-022-02304-2

**Published:** 2022-12-26

**Authors:** Paulene Cay, Cherie A. Singer, Mariam A. Ba

**Affiliations:** Department of Pharmacology/CMM 573, University of Nevada, Reno School of Medicine, 1664 N. Virginia St., Reno, NV 89557-0046 USA

**Keywords:** microRNA, Biomarker, HDM mouse model, Asthma

## Abstract

**Background:**

To date, reliable biomarkers for asthma have not been identified. MicroRNAs (miRNAs) are small, non-coding RNAs that negatively regulate post-transcriptional gene expression, and they are involved in various diseases, including asthma. MiRNAs may serve as ideal biomarkers due to their ability to regulate multiple pathways. This study aims to identify miRNA biomarker signatures for asthma.

**Methods:**

We used the house dust mite (HDM) mouse model of allergic inflammation. Mice were phenotyped by assessing lung function, allergic response, airway inflammation, and remodeling. The miRNA signature profiles in serum and lung tissue were determined by small RNA sequencing, and data were analyzed using Qiagen CLC Genomics Workbench. To identify relevant gene targets, we performed mRNA sequencing, followed by miRNA-targets analysis. These miRNAs and targets were subject to subsequent pathway and functional analyses.

**Results:**

Mice exposed to HDM developed phenotypic features of allergic asthma. miRNA sequencing analysis showed that 213 miRNAs were substantially dysregulated (FDR p-value < 0.05 and fold change expression >  + 1.5 and < − 1.5) in the lung of HDM mice relative to the control mice. In contrast, only one miRNA (miR-146b-5p) was significantly increased in serum. Target analysis of lung dysregulated miRNAs revealed a total of 131 miRNAs targeting 211 mRNAs. Pathway analysis showed T helper 2/1 (Th2/Th1) as the top significantly activated signaling pathway associated with the dysregulated miRNAs. The top enriched diseases were inflammatory response and disease, which included asthma. Asthma network analysis indicated that 113 of 131 miRNAs were directly associated with asthma pathogenesis.

**Conclusions:**

These findings suggest that most dysregulated miRNAs in the HDM model were associated with asthma pathogenesis via Th2 signaling. We identified a panel of 30 miRNAs as potential biomarker candidates for asthma.

**Supplementary Information:**

The online version contains supplementary material available at 10.1186/s12931-022-02304-2.

## Introduction

Asthma is a prevalent chronic inflammatory disease of the airways that affects over 300 million people worldwide and 25 million people in the United States [1]. It is characterized by airway hyper-responsiveness (AHR), remodeling, and smooth muscle cell hyperplasia and hypertrophy [2]. Asthma is classified into multiple phenotypes and endotypes, including allergic, eosinophilic, and neutrophilic with allergic asthma the most prevalent subtype [3]. Heterogeneity in the origin of inflammation contributes to challenges in finding effective treatment and there is no cure or specific biomarkers for asthma. Thus, identifying reliable biomarkers to predict disease pathogenesis would fill a gap in our knowledge that could be useful in therapeutic development.

There is accumulating data supporting a role for microRNAs (miRNAs) as biomarkers for asthma. miRNAs are small, non-coding RNAs that negatively regulate post-transcriptional gene expression via mRNA destabilization or/and degradation [4, 5]. They are involved in various cellular processes such as cell proliferation, differentiation, migration, and apoptosis [6] and are dysregulated in various pathologies, including asthma [7–12]. They are additionally implicated in all key pathogenetic processes of asthma, including airway inflammation, airway hyper-responsiveness (AHR) and hypercontractility. For instance, in a mouse model of allergic asthma, down-regulation of miRNA-133a was associated with increased expression of interleukin 13, and AHR, indicating a potential role of this miRNA in inflammation and airway contractility [13]. Moreover, down-regulation of miRNA-145 alleviated eosinophilic inflammation, mucus hyper-secretion, Th2 production, and AHR [14] in a house dust mite (HDM) model of allergic inflammation. Recent studies have focused on the potential use of miRNAs as biomarkers in various inflammatory diseases, including asthma. For example, in asthma patients, circulating miRNAs were found to be associated with lung function (miR-126-3p, miR-1290, miR-146b-5p, and miR-206), asthma severity (miR-185-5p), and exacerbations (miR-146b-5p, miR-206, and miR-720) [10, 11, 15].

While the use of circulating miRNAs as potential biomarkers for asthma has been investigated, conflicting results that associate circulating miRNAs and asthma have impeded progress ﻿toward finding reliable diagnostic and prognostic biosignatures. For instance, Rodrigo-Muñoz et al., identified a panel of three circulating miRNAs (miR-185-5p, miR-320a, and miR-320b) as biomarkers for asthma [11]. In contrast, another group showed a distinct panel of four miRNAs (miRNA-16-5p, miR-223-3p, miR-570-3p, and miR-299-5p) differentially expressed in asthmatic patients relative to the healthy group and individuals with allergic rhinitis [12]. Subsequent study found a subset of seven miRNAs comprising five miRNA ratios as biomarker signatures for asthma [16].

Another confounding problem is that the expression profiles of miRNAs in response to physiological or pathological stimuli are tissue-specific. Therefore, lung tissue might be a more promising source of biomarker discovery for asthma than circulating miRNAs. In this study, we compared lung miRNA signatures to circulating miRNA profiles using a HDM preclinical model of asthma *to address the hypothesis that the lung contains distinct miRNA signature patterns that can serve as unique biomarkers for asthma.* We have identified a panel of 30 miRNAs as biomarker candidates for asthma. While 18 of the 30 miRNAs had been previously associated with asthma pathogenesis, we have found 12 novel miRNAs (miR-3473b, 7061-5p, 217-5p, 369-3p, 411-3p, 381-3p, miR-7224-3p, 491-5p, 3097-5p, miR-6540-5p, 33-3p, and 1943-5p) correlated with asthma features.

## Materials and methods

### Materials

Sterile endotoxin-free 0.9% saline solution (cat# IAX-900-003) was obtained from Innaxon, UK. House dust mite (HDM, *Dermatophogoides pteronyssinus*, cat # XPB70D3A2.5) extracts were purchased from Stallergenes (Greer Laboratories, Lenoir, NC). Methacholine (acetyl-β-methylcholine chloride) was from Sigma Life Sciences (St. Louis, MO). RNA extraction and cDNA library preparation kits were from QIAGEN (Germantown, MD).

### Methods

#### Mice

Male and female BALB/c mice, aged 6–8 weeks old, were purchased from the Jackson Laboratory and housed in a pathogen-free environment in the laboratory animal medicine facility located in the Center for Molecular Medicine at the University of Nevada, Reno (UNR), School of Medicine. All experimental procedures were approved by the institutional Animal Care and Use Committee (IACUC).

#### HDM sensitization and challenge protocol

The HDM protocol was carried out as previously described [17]. Briefly, mice were sensitized with an intranasal administration of 50 μl of a 25 μg HDM solution or saline once daily for five consecutive days. This was followed by a daily challenge of HDM or saline for five consecutive days per week for 3 weeks.

#### Tracheostomy and lung function assessment

Mice were anesthetized by an intraperitoneal (i.p.) injection of a ketamine (90 mg/kg)/xylazine (10 mg/kg) cocktail. The anesthesia depth was monitored with a toe pinch and heart rate assessment. Mice were laid supine and hair removed from the chest and neck area using commercial hair removal product followed by wet cotton swabs to reduce irritation. The skin, muscles, and fat in the neck were removed, with stainless-steel surgical scissors, to expose the trachea. A small lateral incision was made to the upper trachea, and a surgical tube was inserted 1/3 deep into the trachea above the primary bronchi. The surgical tube was secured into the trachea using a sterile suture. Additional ketamine was often administered after the tracheostomy to ensure proper sedation before lung function assessment. Mice were mechanically ventilated and lung airway resistance and compliance were measured in response to increasing doses (0–12.5 mg/ml in PBS) of aerosolized methacholine using Buxco equipment and FinePoint software from Data Sciences International (St. Paul, MN).

#### Blood collection and serum preparation

Blood was collected after lung function analysis via cardiac puncture as previously described [18] using a 23G needle attached to a 1 ml syringe. After collection, the needle was removed from the syringe, and blood was transferred into serum gel with clotting activator tubes (Sarstedt, Newton, NC). The blood was clotted at room temperature (RT) for 30 min, followed by centrifugation at 2000×*g* for 10 min at 4 °C, and the serum was transferred to a new 1.5 ml tube. The serum was then centrifuged at 16,000×*g* at 4 °C for 10 min to remove additional debris and minimize gDNA contamination. The clarified serum was aliquoted into 200 μl aliquots.

#### Bronchoalveolar lavage and Luminex assays

After the cardiac puncture procedure, bronchoalveolar lavage (BAL) was performed on tracheostomized mice. A total of five lavages were performed per mouse using a 1 ml syringe with a 0.8 ml of sterile 2.6 mM EDTA-saline solution (BAL solution) per lavage. Cell pellets from all five lavages were collected by centrifugation (300×*g* at 4 °C for 5 min), combined into a single tube, resuspended in 500 μ of BAL solution, and subject to cell counting using a Coulter counter (Beckman Coulter Z series, Brea, CA). The supernatants from the first two lavages were pooled and used for Luminex assays. The Luminex multiplex assays were performed at Eve Technologies Corp (Calgary, AB, Canada) using 50 µl BAL fluid per sample with the Mouse Cytokine Array/Chemokine Array 32-Plex Discovery Assay® (MD31) according to the manufacturer's instructions.

#### Lung tissue collection and histology

The right lung lobes and post-caval lobe were collected and placed, at one lobe per tube, into 1 ml tubes containing RNAlater solution (Invitrogen, ThermoFisher Scientific, cat# AM7021, Carlsbad, CA) and stored at − 80 °C for downstream analysis. The left lung lobe was inflated and fixed with 10% neutral buffered formalin (Fisher Scientific, Kalamazoo, MI) at 25 cm above the mouse level. Fixed lungs were embedded in paraffin (FFPE), cut into five μM sections onto positively charged slides. Slides were stained with hematoxylin–eosin (H&E) and Masson’s trichrome according to the Reveal Biosciences’ standard protocol. The whole slide images were obtained using a Panoramic SCAN (3D Histech, Hungary). The quantitative analysis of whole slide images was performed using imageDX software (Reveal Biosciences, San Diego, CA). The inflammatory cells in the H&E-stained lung were determined and quantitated as the number of immune cells within the total image analysis area (mm^2^). The collagen and extracellular matrix (ECM) depositions in the Masson’s Trichrome-stained sections were evaluated as a percent of the entire image analysis area (mm^2^). The representative 20X photos were acquired from whole slide images using the CaseViewer software (3D Histech, Hungary).

#### Detection of serum HDM-specific IgE

HDM-specific IgE in the mouse serum was quantified using the mouse serum anti-HDM IgE antibody assay kit (cat# 3037, Chondrex, Redmond, WA) according to the manufacturer’s recommendations. Briefly, anti-HDM Mouse IgE antibody standards (diluted at a range of 0–50 ng/ml) and mouse serum samples (diluted at 1:10) were added in duplicate wells onto a 96-well ELISA plate precoated with anti-mouse IgE antibody. The plate was incubated for 2 h at RT and washed 3× with wash buffer. The plate was incubated with the biotinylated HDM detection antibody for 2 h at RT and washed 3×. The streptavidin peroxidase was added, incubated at RT for 30 min, and washed 3×. The plate was incubated with TMB (3,3′,5,5′-tetramethylbenzidine) substrate for 25 min at RT, the reaction was stopped with 2N sulfuric acid, and the plate was read at 450 mm.

#### microRNAs sequencing and bioinformatic analysis

microRNA sequencing (miRNA- seq) from serum and lung tissue was performed at the Qiagen Genomic Center (Hilden, Germany) according to the facility's established protocols. For serum samples, RNA was extracted from 200 μl of serum using the miRNeasy serum/plasma advanced kit, and the RNA quality control (QC) was determined by QPCR of serum endogenous miRNAs (such as miRNA-103a-3p and miR-191-5p), hemolysis markers (miR-23a and miR451a), and Spike-Ins control (UniSp6). For lung tissue samples, RNA was extracted with the RNeasy mini kit, quantified using a Nanodrop, and the RNA integrity was assessed using the Agilent TapeStation. miRNA libraries for serum and lung were prepared using Qiagen's miRNA Library Kit following the manufacturer's protocol. cDNA libraries with Unique Molecular Index (UMI) assignment were amplified, cleaned up, QC done by Bioanalyzer, and sequenced using single-end reads, 1 × 75 bp, to a depth of 12–14 million reads (NextSeq 550, Illumina). FASTQ files with quality scores (QS) > 30 were analyzed by CLC Genomics Workbench (version 12.0.2) using the QIAseq miRNA quantification workflow. The de-duplicated Unique Molecular Index (UMI) reads were grouped into unique search sequences and mapped to the miRNAs annotated in the miRbase v22. miRNA expression was calculated via counting the number of distinctive UMI reads corresponding to each miRbase-annotated miRNA and normalized to the sequencing depth (counts per million). Differential miRNA expression between control and HDM-treated mice was assessed using the “Exact Test” for two-group comparisons available as part of the EdgeR Bioconductor package in the CLC Genomic Workbench. The microRNA-Seq data have been deposited at the NCBI Gene Expression Omnibus (https://www.ncbi.nlm.nih.gov/geo) under series accession number GSE214937.

#### mRNA sequencing and bioinformatic analysis

Libraries were prepared from the same lung RNA samples used for miRNA sequencing using QIAseq Stranded mRNA Select Kit with poly-A enrichment. cDNA libraries were amplified, cleaned up, QC performed with a Bioanalyzer, and sequenced using paired-end 2 × 75 bp read, to a depth of 60 million reads (NextSeq 550, Illumina) FASTQ files with quality scores (QS) > 30 were analyzed, mapped, and quantified using the RNA-seq analysis tool from CLC Genomics Workbench (version 21.0.4). Reads were trimmed, mapped to the mouse genome version GRCm38, and annotated using the ENSEMBL Mus_musculus. GRCm38 version 98. Gene expression was calculated by counting number of reads mapping to the annotated gene loci, and the resulting expression values were normalized to transcripts per million (TPM). Differential gene expression between control and HDM-exposed mice was assessed using the “Exact Test” for two-group comparisons available as part of the EdgeR Bioconductor package in the CLC Genomic Workbench. The RNA-Seq data have been deposited at the NCBI Gene Expression Omnibus (https://www.ncbi.nlm.nih.gov/geo) under series accession number GSE214937.

#### miRNA targets identification and pathway analysis

miRNA target identification and pathway analysis were performed using Ingenuity Pathway Analysis (IPA, Qiagen). To determine the most significantly dysregulated molecules, miRNAs and mRNA datasets were filtered using the filter dataset tab on IPA. The cut-off values were log2 fold-change expression (log2 FC) > 0.58 or < − 0.58 and FDR p < 0.05. Next, the mRNA targets were identified using the miRNA target filter. It is known that one miRNA can target up to 200 mRNAs. Therefore, to reduce the list of mRNA targets, we applied filters to include diseases relevant to asthma pathogenesis, such as inflammatory disease, inflammatory response, and respiratory disease. Further, the pathways list was filtered to include those related to a disease-specific pathway, such as airway inflammation in asthma and cellular growth, proliferation, and development pathways (including Th1, Th2, and Th17 activation pathways). The resulting miRNA and mRNA targets were subject to the IPA core analysis [19].

#### cDNA synthesis and QPCR

The lung RNA samples from the same mice used for all the experiments listed above, including pulmonary function, lung histology, and miRNA/RNA sequencing, were utilized to validate the miRNA sequencing data. miRNA cDNA was synthesized from five ng of total RNA using TaqMan Advanced miRNA cDNA Kit (Cat# A28007, Thermofisher Scientific, Waltham, MA) according to the manufacturer’s recommendations. QPCR was performed on the QuantStudio3 Fast Real-time PCR system, 96-well, 0.1 ml plate with 2X Advanced TaqMan Fast Master Mix and specific miRNA probe-primer mix. miRNA expression was normalized to mmu-miR5121. The miRNA mmu-miR-5121 was chosen as reference miRNA because its expression in the dataset remains unchanged in response to HDM exposure.

#### Statistics

Mice characterization data were graphed and analyzed using Graph Pad Prism software. Data represent mean ± standard error mean (SEM). An unpaired t-test with Welch's correction was used for two-group comparison and p ≤ 0.05 is considered statistically significant. For the miRNA and RNA sequencing data, the false discovery rate (FDR) p-values were calculated using the Benjamini–Hochberg method to account for multiple testing issues, and FDR < 0.05 was considered significant. The networks, pathways, and functional analyses were done through IPA. Right-tailed Fisher’s Exact Test was used to calculate p-values, and p < 0.05 (− log_10_ p-value > 1.3) was considered significant.

## Results

### HDM exposure increases airway resistance and induces allergic inflammatory response

**Fig. 1:**
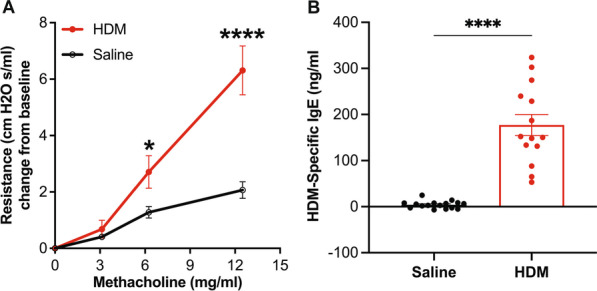
Assessment of airway hyper-responsiveness and IgE production. **A** Airway resistance was measured in response to increasing doses of methacholine, and **B** serum HDM-specific IgE was measured using ELISA. The graphs represent mean ± SEM, n = 16–18, *p < 0.05 and ****p < 0.0001 HDM-challenged mice versus alum control, two-way ANOVA followed by Dunnett's multiple comparisons (**A**) or unpaired t-test with Welch's correction (**B**)

**Fig. 2:**
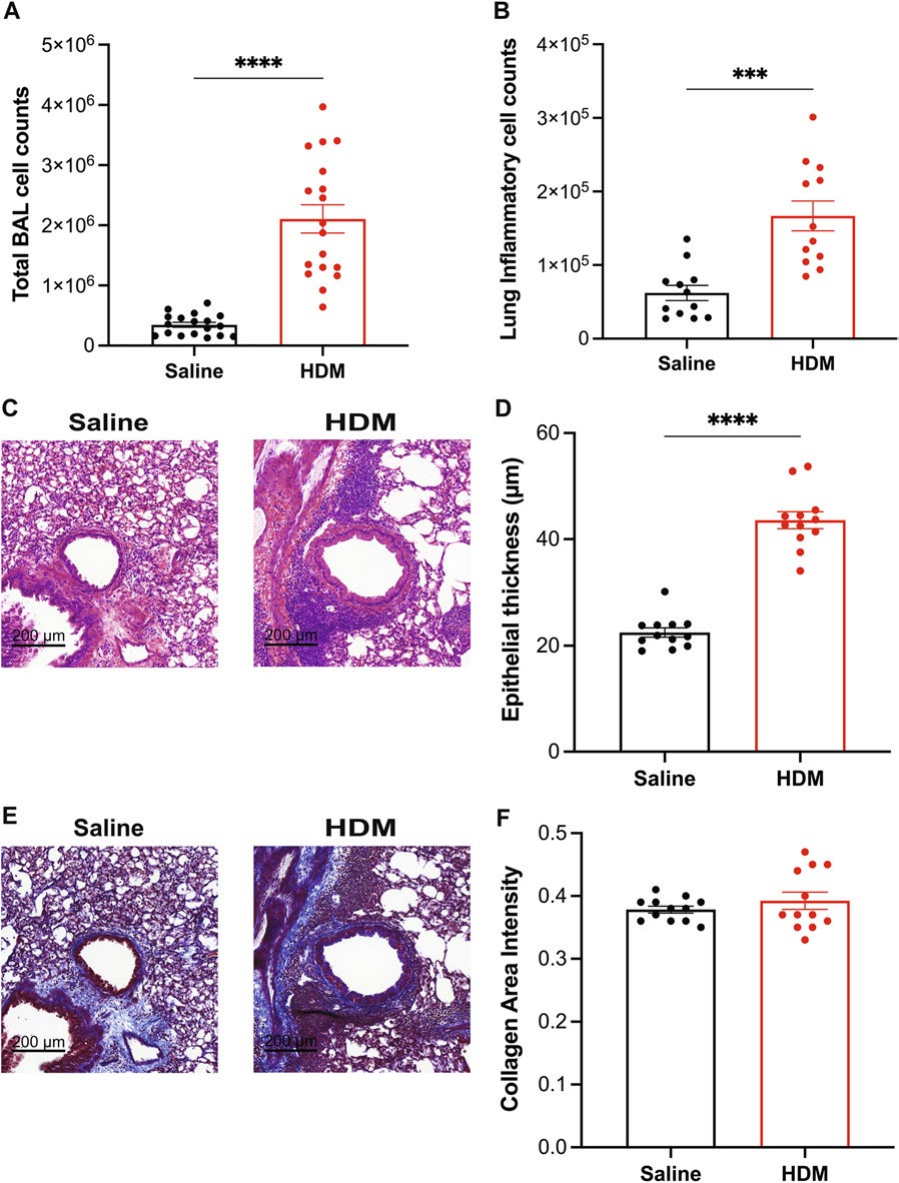
Total inflammatory cell counts in Bronchioloalveolar lavage (BAL) and lung histology. **A** Total BAL cell counts were performed by coulter counting. **C** and **E** are representative images of lung sections stained with hematoxylin and eosin (**C**), and mason trichrome (**E**). **B** and **D** are quantification of inflammatory cell count (**B**) and epithelial airway thickness (**D**) in H&E-stained lungs. **F** The quantification of collagen deposition in mason trichrome-stained lung sections. Data represent mean ± SEM, n = 12–18 per group, ***p < 0.001 and ****p < 0.0001 HDM-challenged mice relative alum control using unpaired t-test with Welch's correction

To confirm the efficacy of HDM exposure on lung function, mice were anesthetized with ketamine/xylazine, tracheostomized, and airway resistance was measured in response to increasing doses of methacholine (0–12.5 mg/ml). HDM sensitized and challenged mice displayed a significantly increased lung resistance compared to the controls (Fig. 1A). These mice also showed a substantial elevation in the concentration of serum HDM-specific IgE relative to the saline controls (Fig. 1B). To analyze the effect of HDM sensitization and challenge on inflammation and remodeling, we assessed the total inflammatory cell count in the BAL fluid and performed histology in FFPE lung sections. The total BAL inflammatory cell counts were significantly higher in mice exposed to HDM compared to controls (Fig. 2A). Hematoxylin–eosin (H&E) staining revealed that HDM mice had a substantial increase in lung inflammation and thicker airways than the saline controls (Fig. 2B–D). However, the collagen deposition levels were unchanged between the two groups (Fig. 2E, F). These results suggest that HDM sensitization and challenge trigger airway inflammation and remodeling but do not affect ECM deposition.

**Fig. 3:**
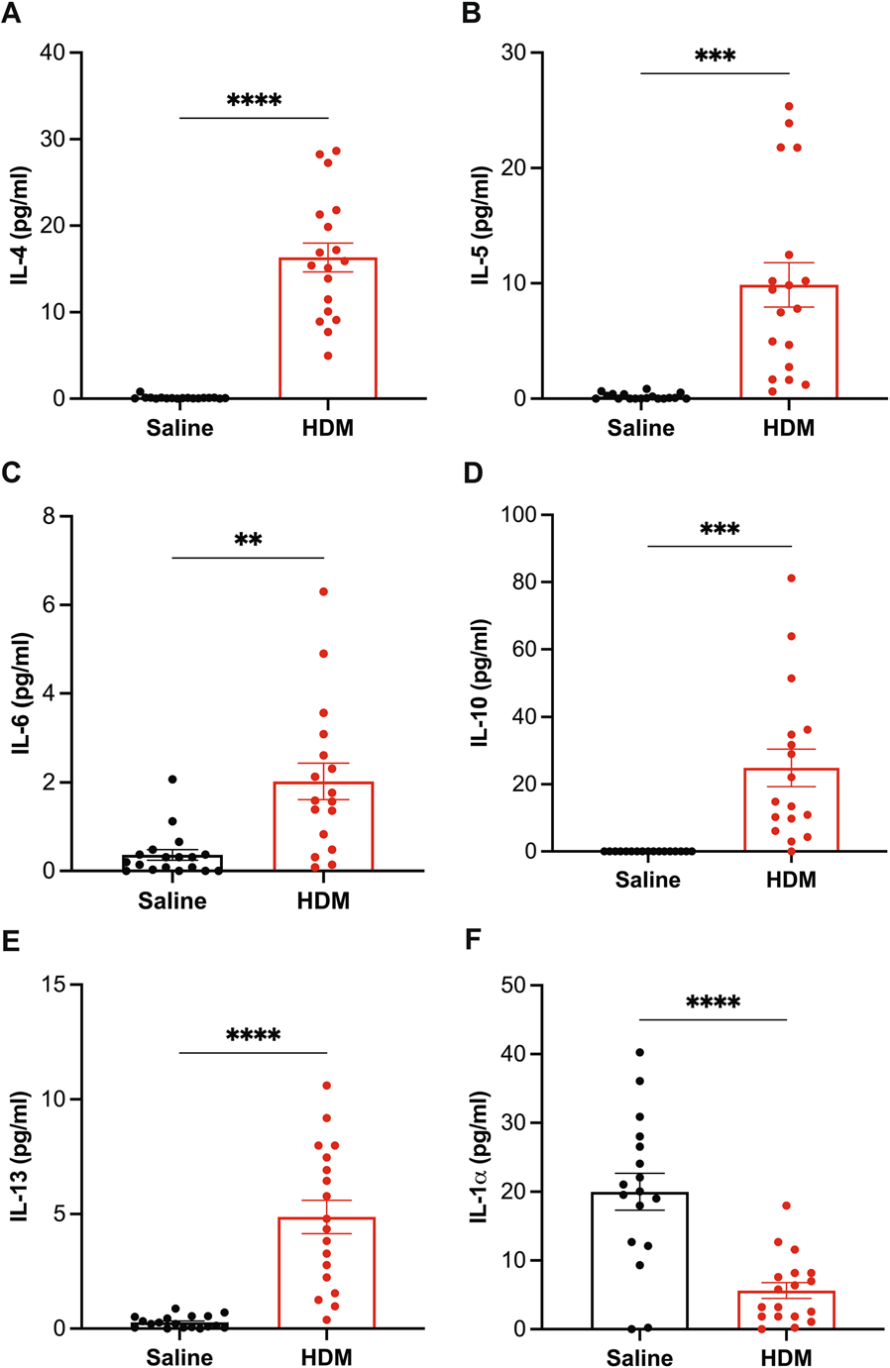
Quantification of cytokine production in the BAL fluid. The cytokine secretion in BAL fluid was analyzed via Luminex assays. **A** IL-4, **B** IL-5, **C** IL-6, **D** IL-10, **E** IL-13 and **F** IL-1α secretion levels. Data are expressed as mean ± SEM, n = 16–18, **p < 0.01 ***p < 0.001 and ****p < 0.0001 versus alum control, unpaired t-test with Welch's correction

**Fig. 4:**
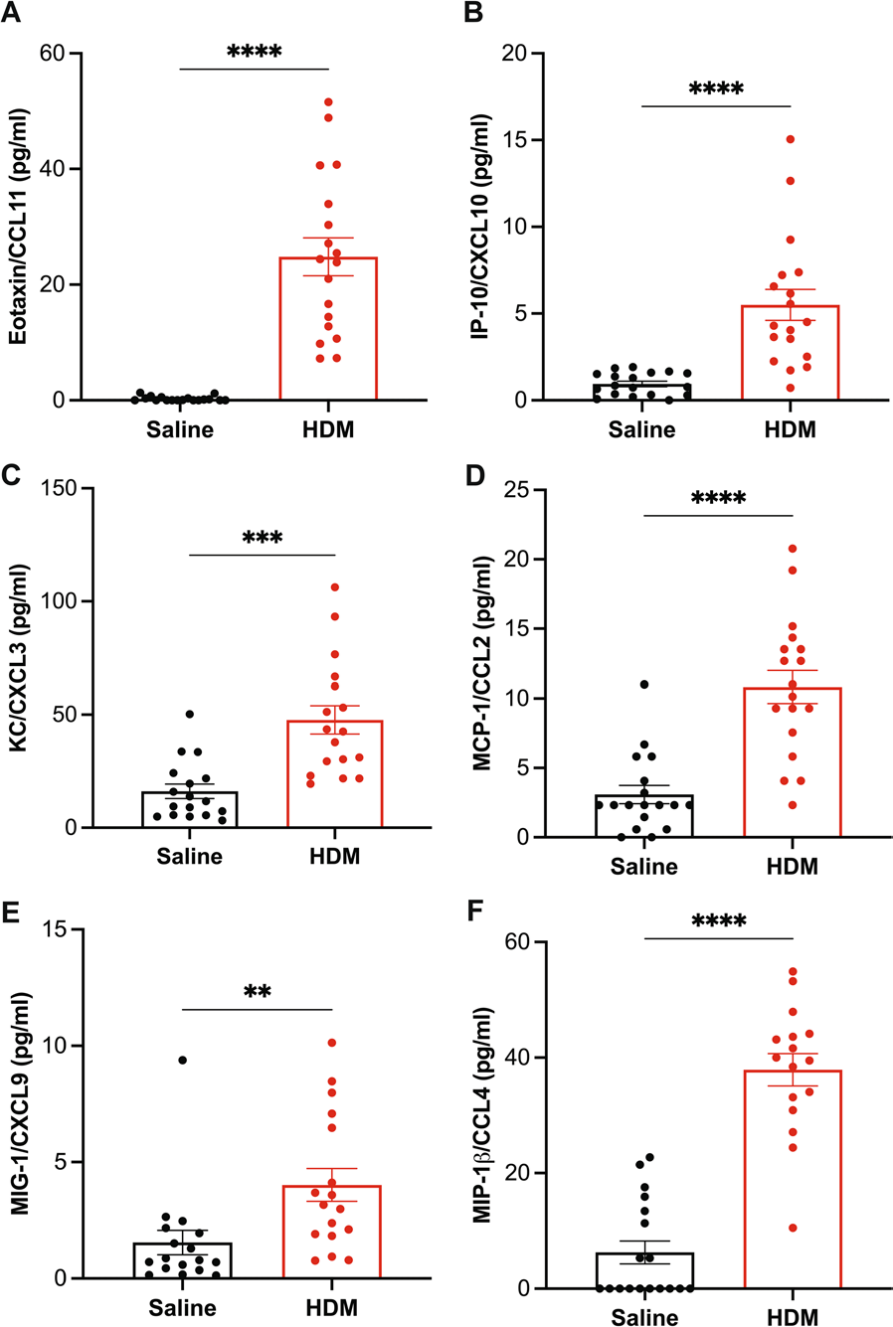
Quantification of chemokine secretion in the BAL fluid. The inflammatory chemokine secretion in BAL fluid was analyzed via Luminex assays. **A** Eotaxin/CCL11, **B** IP-10/CXCL10, **C** KC/CXCL3, **D** MCP-1/CCL2, **E** MIG/CXCL9 and **F** MIP-1β/CCL4, **A**–**F** chemokine quantities. Data are expressed as mean ± SEM, n = 16–18, **p < 0.01 ***p < 0.001 and ****p < 0.0001 versus alum control, unpaired t-test with Welch's correction

The effect of HDM sensitization and challenge on various inflammatory cytokines and chemokines was assessed via Luminex assays. The secretion of major Th2 cytokines, IL-4, 5, 6, 10, and 13, were significantly enhanced in HDM mice compared to the controls (Fig. 3A–E). In contrast, IL-1α levels were reduced in HDM mice relative to controls (Fig. 3F). Additionally, the production levels of chemokines, including eotaxin/CCL11, IP-10/CXCL10, KC/CXCL3, MCP-1/CCL2, MIG/CXCL9 and MIP-1β/CCL4, and were significantly higher in BAL fluid of HDM mice relative to saline controls (Fig. 4). As expected, the HDM model exhibited increased production of key inflammatory mediators. Taken together, these results validate the use of the HDM model for these studies. We also analyzed the phenotypic features of the HDM mouse model based on sex and found that female HDM-exposed mice exhibited a similar allergic inflammatory profile as the male HDM counterparts (data not shown). Therefore, both sexes were combined for all subsequent analyses.

### Dysregulation of miRNA expression in the HDM mouse model of allergic inflammation

**Fig. 5:**
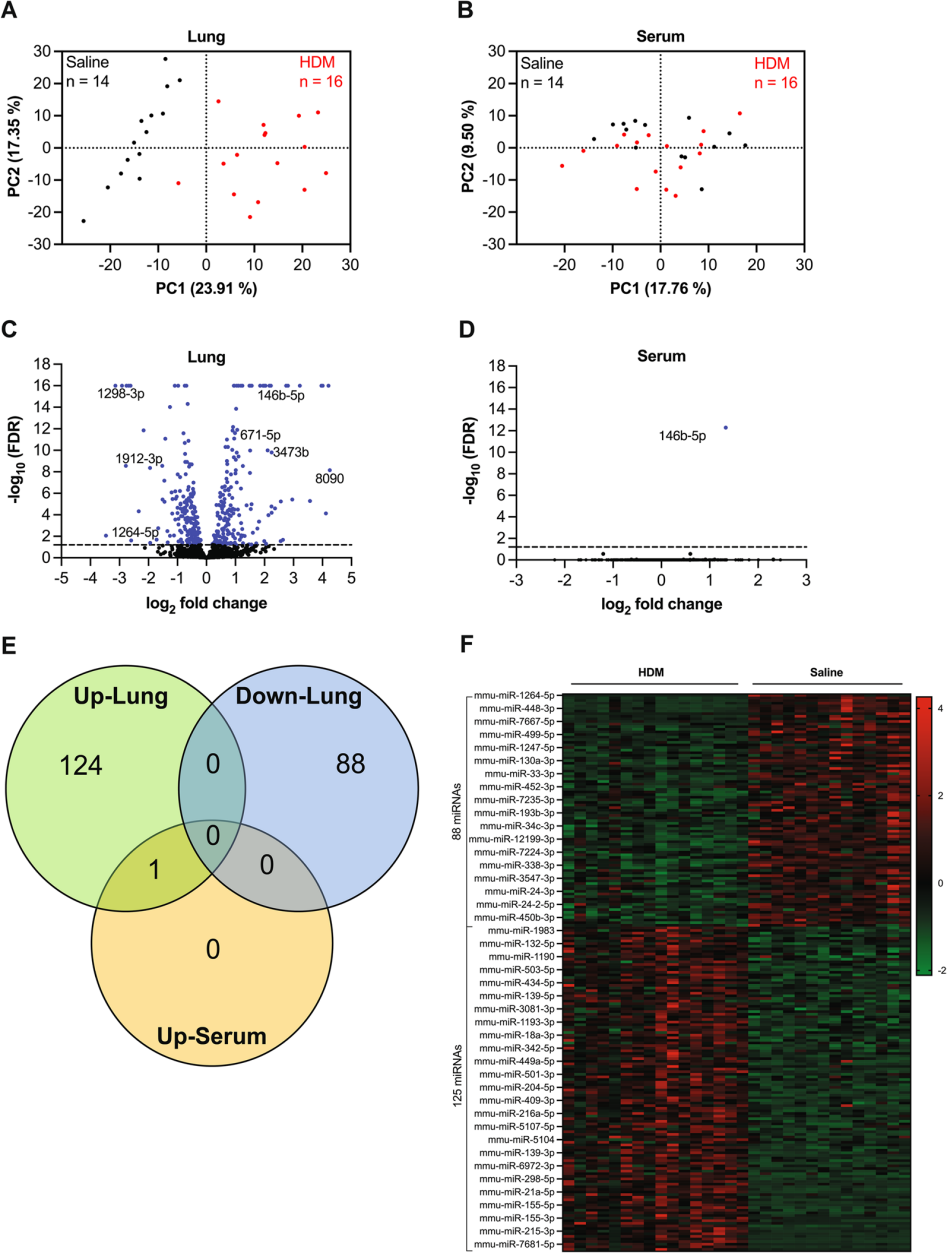
miRNA signature profiles in the house dust mite (HDM) mouse model of allergic inflammation. **A** Principal component analysis (PCI) of lung samples, **B** PCI of serum samples, red dots represent mice exposed to HDM, and black dots are control mice. **C**, **D** Volcano plots of differentially perturbed miRNAs in the lung tissue (**C**) and serum samples (**D**), red dots indicate the significantly dysregulated miRNAs (FDR < 0.05), dotted line show the significance threshold (− log_10_ (FDR) = 1.3). A − log_10_ (FDR) > 1.3 is considered as statistically significant. **E** Venn diagram of significantly up-regulated miRNAs in lung (green circle), serum (orange circle) samples, and down-regulated miRNAs in lung (blue circle). The miRNAs included in this analysis have a log2 fold change expression cut off >  + 0.58 and cut off < − 0.58 in HDM vs saline mice. n = 14–16 per group, and FDR < 0.05 is considered statistically significant. The Venn diagram was designed using the online Venny 2.1.0, https://bioinfogp.cnb.csic.es/tools/venny/index.html, with slight modifications. **F** Heat map of the 213 miRNAs that were substantially dysregulated in the HDM mice vs. saline controls. Red squares show upregulated miRNAs and green represent downregulated miRNAs. The heat map was generated by scaling the data using Z-scores of each miRNA (normalized CPM were converted into z-scores)

Principal component analysis (PCA) of miRNA expression in lung samples showed two nicely separated clusters between mice treated with saline and HDM (Fig. 5A). In contrast, there was no clear clustering observed between saline and HDM mice in serum samples (Fig. 5B). In the lung tissue, a total of 345 miRNAs were significantly differentially expressed (FDR < 0.05 or − log_10_ (FDR) > 1.3) in HDM mice relative to control (Fig. 5C), but only one miRNA (miR-146b-5p) was substantially upregulated (FDR < 0.05 or − log_10_ (FDR) > 1.3) in serum (Fig. 5D, E). Thus, the rest of this study focuses on lung miRNA signatures. Further analysis of the lung miRNA profile indicated that 125 miRNAs were up-regulated and 88 miRNAs were downregulated by at least 1.5-fold change in HDM mice compared to saline control (Fig. 5E, F). This lung miRNA signature was the focus of the rest of this study (Fig. 5F and Table 1).

**Table 1:** Fold change and FDR values of dysregulated microRNAs

miRNA ID	miRNA Symbol	Log2 FC	FDR
mmu-miR-8090	miR-8090 (miRNAs w/seed AAGCGCA)	4.253	7.13E−09
mmu-miR-135b-3p	miR-135b-3p (and other miRNAs w/seed UGUAGGG)	4.207	0.00E+00
mmu-miR-7681-5p	miR-3058-3p (and other miRNAs w/seed UCCUGUC)	4.121	7.36E-05
mmu-miR-135b-5p	miR-135a-5p (and other miRNAs w/seed AUGGCUU)	4.006	0.00E+00
mmu-miR-147-3p	miR-147 (and other miRNAs w/seed UGUGCGG)	3.961	0.00E+00
mmu-miR-7062-5p	miR-7062-5p (and other miRNAs w/seed GGAGGCC)	3.569	5.12E-06
mmu-miR-147-5p	miR-147-5p (and other miRNAs w/seed GGAAACA)	3.222	0.00E+00
mmu-miR-215-3p	miR-215-3p (miRNAs w/seed CUGUCAU)	2.956	3.75E−06
mmu-miR-215-5p	miR-192-5p (and other miRNAs w/seed UGACCUA)	2.809	0.00E+00
mmu-miR-6978-3p	miR-6978-3p (miRNAs w/seed CGGCUUC)	2.747	0.00E+00
mmu-miR-7015-5p	miR-7015-5p (miRNAs w/seed CUGUGCA)	2.637	2.11E−02
mmu-miR-1948-3p	miR-1948-3p (miRNAs w/seed UUAGGCA)	2.566	5.80E−06
mmu-miR-155-3p	miR-155-3p (miRNAs w/seed UCCUACC)	2.559	2.79E−02
mmu-miR-7678-3p	miR-7678-3p (miRNAs w/seed UUCCUUA)	2.365	2.50E−05
mmu-miR-292b-5p	miR-292b-5p (and other miRNAs w/seed CUCAAAA)	2.25	6.32E−05
mmu-miR-7676-3p	miR-7676-3p (miRNAs w/seed CCGGUGC)	2.249	1.59E−10
mmu-miR-1948-5p	miR-1948-5p (miRNAs w/seed UAUGAGU)	2.241	1.69E-05
mmu-miR-155-5p	miR-155-5p (miRNAs w/seed UAAUGCU)	2.217	0.00E+00
mmu-miR-296-3p	miR-296-3p (miRNAs w/seed AGGGUUG)	2.168	0.00E+00
mmu-miR-2137	miR-2137 (miRNAs w/seed CCGGCGG)	2.134	1.08E−04
mmu-miR-3473b	miR-3473b (and other miRNAs w/seed GGCUGGA)	2.109	9.89E−11
mmu-miR-5120	miR-4260 (and other miRNAs w/seed UUGGGGC)	2.051	4.92E-02
mmu-miR-21a-5p	miR-21-5p (and other miRNAs w/seed AGCUUAU)	2.035	0.00E+00
mmu-miR-21a-3p	miR-21-3p (and other miRNAs w/seed AACAGCA)	2.028	0.00E+00
mmu-miR-148a-5p	miR-148a-5p (and other miRNAs w/seed AAGUUCU)	1.995	0.00E+00
mmu-miR-6978-5p	miR-6782-5p (and other miRNAs w/seed AGGGGUG)	1.992	2.82E−02
mmu-miR-138-5p	miR-138-5p (miRNAs w/seed GCUGGUG)	1.982	0.00E+00
mmu-miR-298-5p	miR-298-5p (and other miRNAs w/seed GCAGAGG)	1.954	0.00E+00
mmu-miR-511-3p	miR-511-3p (miRNAs w/seed AUGUGUA)	1.853	0.00E+00
mmu-miR-1945	miR-1945 (miRNAs w/seed CUUCGCG)	1.812	4.30E−04
mmu-miR-7061-5p	miR-3688-3p (and other miRNAs w/seed AUGGAAA)	1.76	1.87E−03
mmu-miR-1938	miR-1938 (miRNAs w/seed GGUGGGA)	1.719	4.54E−02
mmu-miR-6972-3p	miR-6972-3p (miRNAs w/seed UACUGUA)	1.6	4.73E−03
mmu-miR-146b-5p	miR-146a-5p (and other miRNAs w/seed GAGAACU)	1.561	0.00E+00
mmu-miR-704	miR-704 (miRNAs w/seed GACAUGU)	1.558	1.02E−05
mmu-miR-7091-3p	miR-7091-3p (miRNAs w/seed GUGGCUU)	1.53	2.89E−05
mmu-miR-148a-3p	miR-148a-3p (and other miRNAs w/seed CAGUGCA)	1.528	0.00E+00
mmu-miR-139-3p	miR-139-3p (miRNAs w/seed GGAGACG)	1.507	1.05E−10
mmu-miR-665-3p	miR-665 (and other miRNAs w/seed CCAGGAG)	1.503	1.27E−08
mmu-miR-511-5p	miR-511-5p (miRNAs w/seed UGCCUUU)	1.484	0.00E+00
mmu-miR-370-3p	miR-370-3p (and other miRNAs w/seed CCUGCUG)	1.387	1.31E−05
mmu-miR-3062-5p	miR-3062-5p (miRNAs w/seed GAGAAUG)	1.319	9.27E−03
mmu-miR-5104	miR-5104 (miRNAs w/seed UGUGCUA)	1.265	8.58E−03
mmu-miR-204-3p	miR-204-3p (and other miRNAs w/seed CUGGGAA)	1.261	4.37E−02
mmu-miR-376a-3p	miR-376a-3p (miRNAs w/seed UCGUAGA)	1.258	2.83E−09
mmu-miR-362-5p	miR-362-5p (and other miRNAs w/seed AUCCUUG)	1.239	0.00E+00
mmu-miR-138-1-3p	miR-138-1-3p (miRNAs w/seed GGCUACU)	1.237	2.10E−03
mmu-miR-5107-5p	miR-5107-5p (miRNAs w/seed GGGCAGA)	1.205	8.02E−03
mmu-miR-217-5p	miR-217-5p (and other miRNAs w/seed ACUGCAU)	1.204	1.13E−02
mmu-miR-18b-5p	miR-18a-5p (and other miRNAs w/seed AAGGUGC)	1.193	3.58E−07
mmu-miR-146b-3p	miR-146b-3p (miRNAs w/seed CCCUAGG)	1.19	0.00E+00
mmu-miR-677-5p	miR-4276 (and other miRNAs w/seed UCAGUGA)	1.151	2.11E−02
mmu-miR-216a-5p	miR-216a-5p (miRNAs w/seed AAUCUCA)	1.129	1.06E−04
mmu-miR-142a-3p	miR-142-3p (and other miRNAs w/seed GUAGUGU)	1.109	3.71E−10
mmu-miR-7a-5p	miR-7a-5p (and other miRNAs w/seed GGAAGAC)	1.106	0.00E+00
mmu-miR-449a-3p	miR-449a-3p (miRNAs w/seed AGCUAAC)	1.09	5.74E−03
mmu-miR-341-3p	miR-341 (and other miRNAs w/seed CGGUCGA)	1.085	3.21E−05
mmu-miR-409-3p	miR-409-3p (miRNAs w/seed AAUGUUG)	1.061	1.25E−12
mmu-miR-369-3p	miR-369-3p (miRNAs w/seed AUAAUAC)	1.049	9.66E−08
mmu-miR-378c	miR-378a-3p (and other miRNAs w/seed CUGGACU)	1.049	0.00E+00
mmu-miR-501-5p	miR-501-5p (miRNAs w/seed AUCCUUU)	1.048	1.65E−10
mmu-miR-5099	miR-5099 (miRNAs w/seed UAGAUCG)	1.024	1.42E−14
mmu-miR-204-5p	miR-204-5p (and other miRNAs w/seed UCCCUUU)	1.011	7.05E−09
mmu-miR-130b-5p	miR-130b-5p (and other miRNAs w/seed CUCUUUC)	1.003	1.41E−08
mmu-miR-671-5p	miR-671-5p (miRNAs w/seed GGAAGCC)	0.984	2.49E−12
mmu-miR-3110-5p	miR-3110-5p (and other miRNAs w/seed UCUGCCU)	0.984	1.57E−02
mmu-miR-7013-5p	miR-7013-5p (miRNAs w/seed AUGAAGA)	0.978	4.02E−02
mmu-miR-501-3p	miR-501-3p (and other miRNAs w/seed AUGCACC)	0.967	3.27E−05
mmu-miR-335-3p	miR-335-3p (miRNAs w/seed UUUUCAU)	0.957	8.04E−12
mmu-miR-340-5p	miR-340-5p (miRNAs w/seed UAUAAAG)	0.95	4.50E−10
mmu-miR-205-5p	miR-205-5p (and other miRNAs w/seed CCUUCAU)	0.945	2.70E−03
mmu-miR-378a-5p	miR-378a-5p (miRNAs w/seed UCCUGAC)	0.945	0.00E+00
mmu-miR-449a-5p	miR-34a-5p (and other miRNAs w/seed GGCAGUG)	0.929	7.45E−05
mmu-miR-540-5p	miR-540-5p (miRNAs w/seed AAGGGUC)	0.926	9.95E−03
mmu-miR-379-5p	miR-379-5p (and other miRNAs w/seed GGUAGAC)	0.918	6.75E−13
mmu-miR-409-5p	miR-409-5p (and other miRNAs w/seed GGUUACC)	0.911	2.73E−05
mmu-miR-666-3p	miR-666-3p (miRNAs w/seed GCUGCAG)	0.904	6.09E−03
mmu-miR-342-5p	miR-342-5p (and other miRNAs w/seed GGGGUGC)	0.89	9.09E−11
mmu-miR-3473a	miR-185-5p (and other miRNAs w/seed GGAGAGA)	0.89	2.66E−06
mmu-miR-1949	miR-1949 (and other miRNAs w/seed UAUACCA)	0.858	4.43E−02
mmu-miR-378b	miR-378b (and other miRNAs w/seed UGGACUU)	0.851	4.33E−05
mmu-miR-299a-3p	miR-299a-3p (and other miRNAs w/seed AUGUGGG)	0.85	1.73E−08
mmu-miR-18a-3p	miR-18a-3p (and other miRNAs w/seed CUGCCCU)	0.845	2.63E−07
mmu-miR-679-5p	miR-679-5p (and other miRNAs w/seed GACUGUG)	0.842	4.08E−02
mmu-miR-9-3p	miR-9-3p (and other miRNAs w/seed UAAAGCU)	0.841	1.29E−04
mmu-miR-362-3p	miR-329-3p (and other miRNAs w/seed ACACACC)	0.836	1.33E−05
mmu-miR-5107-3p	miR-5107-3p (and other miRNAs w/seed AACCUGU)	0.792	1.03E−04
mmu-miR-1193-3p	miR-370-5p (and other miRNAs w/seed AGGUCAC)	0.791	1.38E−06
mmu-miR-212-5p	miR-212-5p (miRNAs w/seed CCUUGGC)	0.791	3.07E−05
mmu-miR-7654-5p	miR-7654-5p (miRNAs w/seed GAGUCGC)	0.772	8.05E−04
mmu-miR-9-5p	miR-9-5p (and other miRNAs w/seed CUUUGGU)	0.766	5.18E−07
mmu-miR-5103	miR-5103 (miRNAs w/seed CAUCCGG)	0.765	4.50E−02
mmu-miR-3081-3p	miR-3081-3p (miRNAs w/seed UGCGCUC)	0.764	3.89E−03
mmu-miR-376b-3p	miR-376a-3p (and other miRNAs w/seed UCAUAGA)	0.762	2.53E−04
mmu-miR-369-5p	miR-369-5p (miRNAs w/seed GAUCGAC)	0.759	4.05E−07
mmu-miR-7091-5p	miR-7091-5p (miRNAs w/seed UAGGGGU)	0.752	5.91E−03
mmu-miR-376a-5p	miR-376a-5p (and other miRNAs w/seed GUAGAUU)	0.748	4.75E−02
mmu-miR-139-5p	miR-139-5p (miRNAs w/seed CUACAGU)	0.741	4.93E−11
mmu-miR-411-3p	miR-411-3p (and other miRNAs w/seed AUGUAAC)	0.74	6.43E−03
mmu-miR-5123	miR-5123 (miRNAs w/seed GUAGAUC)	0.735	4.31E−05
mmu-miR-296-5p	miR-296-5p (miRNAs w/seed GGGCCCC)	0.731	6.02E−04
mmu-miR-574-5p	miR-574-5p (miRNAs w/seed GAGUGUG)	0.72	2.72E−03
mmu-miR-434-5p	miR-434-5p (miRNAs w/seed CUCGACU)	0.719	9.09E−08
mmu-miR-134-5p	miR-3118 (and other miRNAs w/seed GUGACUG)	0.718	1.87E−09
mmu-miR-142a-5p	miR-142-5p (and other miRNAs w/seed AUAAAGU)	0.717	4.17E−04
mmu-miR-223-5p	miR-223-5p (miRNAs w/seed GUGUAUU)	0.709	6.45E−05
mmu-miR-411-5p	miR-411-5p (and other miRNAs w/seed AGUAGAC)	0.708	1.01E−08
mmu-miR-503-5p	miR-503-5p (miRNAs w/seed AGCAGCG)	0.706	1.00E−11
mmu-miR-378d	miR-3176 (and other miRNAs w/seed CUGGCCU)	0.706	9.87E−10
mmu-miR-494-3p	miR-494-3p (miRNAs w/seed GAAACAU)	0.692	4.43E−08
mmu-miR-879-5p	miR-879-5p (and other miRNAs w/seed GAGGCUU)	0.689	1.67E−02
mmu-miR-3473d	miR-674-5p (and other miRNAs w/seed CACUGAG)	0.673	1.09E−03
mmu-miR-1190	miR-1190 (miRNAs w/seed CAGCUGA)	0.668	1.64E−02
mmu-miR-323-3p	miR-323-3p (and other miRNAs w/seed ACAUUAC)	0.661	3.40E−02
mmu-miR-466a-3p	miR-297a-3p (and other miRNAs w/seed AUACAUA)	0.648	2.02E−04
mmu-miR-329-5p	miR-329-5p (miRNAs w/seed GAGGUUU)	0.646	7.53E−05
mmu-miR-154-5p	miR-154-5p (miRNAs w/seed AGGUUAU)	0.641	5.32E−07
mmu-miR-541-5p	miR-541-5p (miRNAs w/seed AGGGAUU)	0.638	2.25E−07
mmu-miR-132-5p	miR-132-5p (miRNAs w/seed ACCGUGG)	0.624	3.41E−06
mmu-miR-540-3p	miR-540-3p (and other miRNAs w/seed GGUCAGA)	0.616	1.20E−02
mmu-miR-3074-5p	miR-3074-5p (miRNAs w/seed UUCCUGC)	0.615	2.79E−02
mmu-miR-381-3p	miR-381-3p (and other miRNAs w/seed AUACAAG)	0.614	7.24E−06
mmu-miR-3105-5p	miR-3105-5p (miRNAs w/seed GAGCAAG)	0.609	3.11E−04
mmu-miR-1983	miR-1983 (and other miRNAs w/seed UCACCUG)	0.605	1.75E−03
mmu-miR-127-5p	miR-127-5p (miRNAs w/seed UGAAGCU)	0.598	1.49E−05
mmu-miR-1258-3p	miR-1258-3p (miRNAs w/seed UAGGGAA)	− 0.582	4.36E−02
mmu-miR-187-3p	miR-187-3p (miRNAs w/seed CGUGUCU)	− 0.598	3.27E−09
mmu-miR-7048-3p	miR-7048-3p (and other miRNAs w/seed CUUCCAU)	− 0.6	1.71E−02
mmu-miR-450b-3p	miR-450a-1-3p (and other miRNAs w/seed UUGGGAA)	− 0.611	2.78E−06
mmu-miR-6911-3p	miR-4705 (and other miRNAs w/seed CAAUCAC)	− 0.614	3.48E−03
mmu-miR-674-3p	miR-674-3p (miRNAs w/seed ACAGCUC)	− 0.615	2.58E−07
mmu-miR-6964-3p	miR-578 (and other miRNAs w/seed UUCUUGU)	− 0.62	2.22E−02
mmu-miR-24-2-5p	miR-24-1-5p (and other miRNAs w/seed UGCCUAC)	− 0.628	6.80E−03
mmu-miR-125a-5p	miR-125b-5p (and other miRNAs w/seed CCCUGAG)	− 0.633	4.76E−07
mmu-miR-193a-5p	miR-193a-5p (miRNAs w/seed GGGUCUU)	− 0.637	3.91E−04
mmu-miR-12202-3p	miR-12202-3p (and other miRNAs w/seed CUUCUCU)	− 0.643	4.31E−02
mmu-miR-7063-3p	miR-6973b-3p (and other miRNAs w/seed GCUCUCU)	− 0.645	2.88E−02
mmu-miR-24-3p	miR-24-3p (and other miRNAs w/seed GGCUCAG)	− 0.647	5.01E−15
mmu-miR-486a-5p	miR-486-5p (and other miRNAs w/seed CCUGUAC)	− 0.65	3.55E−03
mmu-miR-328-3p	miR-328-3p (and other miRNAs w/seed UGGCCCU)	− 0.657	7.86E−03
mmu-miR-7054-3p	miR-7054-3p (miRNAs w/seed CCAACUU)	− 0.66	2.10E−02
mmu-miR-676-5p	miR-519a-2-5p (and other miRNAs w/seed CUCUACA)	− 0.668	1.21E−05
mmu-miR-3547-3p	miR-3547 (and other miRNAs w/seed GAGCACC)	− 0.671	2.54E−02
mmu-miR-29a-5p	miR-29a-5p (miRNAs w/seed CUGAUUU)	− 0.674	4.37E−05
mmu-miR-455-3p	miR-455-3p (miRNAs w/seed CAGUCCA)	− 0.68	6.98E−06
mmu-miR-7083-5p	miR-12197-3p (and other miRNAs w/seed CGGGGCU)	− 0.682	2.56E−02
mmu-miR-7075-3p	miR-7075-3p (miRNAs w/seed AACCAUG)	− 0.683	5.31E−04
mmu-miR-338-3p	miR-338-3p (miRNAs w/seed CCAGCAU)	− 0.685	4.17E−06
mmu-miR-23a-3p	miR-23a-3p (and other miRNAs w/seed UCACAUU)	− 0.692	0.00E+00
mmu-miR-195a-3p	miR-195-3p (and other miRNAs w/seed CAAUAUU)	− 0.712	1.67E−08
mmu-miR-6946-3p	miR-4659a-3p (and other miRNAs w/seed UUCUUCU)	− 0.723	2.13E−04
mmu-miR-32-3p	miR-32-3p (miRNAs w/seed AAUUUAG)	− 0.725	2.73E−04
mmu-miR-7224-3p	miR-181a-2-3p (and other miRNAs w/seed CCACUGA)	− 0.728	2.20E−03
mmu-miR-491-5p	miR-491-5p (and other miRNAs w/seed GUGGGGA)	− 0.731	2.16E−05
mmu-miR-6412	miR-6412 (miRNAs w/seed CGAAACC)	− 0.733	1.22E−09
mmu-miR-151-5p	miR-151-5p (and other miRNAs w/seed CGAGGAG)	− 0.738	2.19E−10
mmu-miR-145b	miR-145-5p (and other miRNAs w/seed UCCAGUU)	− 0.741	4.11E−05
mmu-miR-12199-3p	miR-12199-3p (and other miRNAs w/seed CACUGGU)	− 0.741	7.36E−05
mmu-miR-181a-2-3p	miR-181a-2-3p (miRNAs w/seed CCACCGA)	− 0.746	2.03E−11
mmu-miR-26a-5p	miR-26a-5p (and other miRNAs w/seed UCAAGUA)	− 0.747	0.00E+00
mmu-miR-3968	miR-3968 (miRNAs w/seed GAAUCCC)	− 0.755	6.73E−06
mmu-miR-3097-3p	miR-3097-3p (miRNAs w/seed UCAGACC)	− 0.783	2.42E−05
mmu-miR-34c-3p	miR-34c-3p (and other miRNAs w/seed AUCACUA)	− 0.799	1.97E−06
mmu-miR-5100	miR-5100 (miRNAs w/seed CGAAUCC)	− 0.824	5.14E−05
mmu-miR-181d-3p	miR-181d-3p (miRNAs w/seed CCACCGG)	− 0.83	1.32E−04
mmu-miR-3083-5p	miR-3083-5p (and other miRNAs w/seed GGCUGGG)	− 0.831	3.78E−02
mmu-miR-7052-3p	miR-7052-3p (miRNAs w/seed CUCUGCC)	− 0.834	3.68E−02
mmu-miR-193b-3p	miR-193a-3p (and other miRNAs w/seed ACUGGCC)	− 0.857	4.34E−03
mmu-miR-7008-3p	miR-7008-3p (miRNAs w/seed GUGCUUC)	− 0.873	1.34E−04
mmu-miR-486b-3p	miR-486-3p (and other miRNAs w/seed GGGGCAG)	− 0.881	1.99E−02
mmu-miR-101a-5p	miR-101a-5p (miRNAs w/seed CAGUUAU)	− 0.886	2.69E−05
mmu-miR-130a-5p	miR-130a-5p (miRNAs w/seed CUCUUUU)	− 0.889	9.11E−06
mmu-miR-7235-3p	miR-3583-3p (and other miRNAs w/seed CUGACUU)	− 0.898	1.10E−05
mmu-miR-193b-5p	miR-193b-5p (miRNAs w/seed GGGGUUU)	− 0.904	2.67E−02
mmu-miR-676-3p	miR-676 (and other miRNAs w/seed CGUCCUG)	− 0.906	3.07E−08
mmu-miR-3097-5p	miR-3097-5p (and other miRNAs w/seed ACAGGUG)	− 0.908	3.02E−03
mmu-miR-1668	miR-1668 (and other miRNAs w/seed AAAGGCC)	− 0.913	6.01E−07
mmu-miR-452-3p	miR-452-3p (miRNAs w/seed CAGUCUC)	− 0.964	8.07E−04
mmu-miR-384-5p	miR-30c-5p (and other miRNAs w/seed GUAAACA)	− 0.964	3.05E−02
mmu-miR-6540-5p	miR-5624-3p (and other miRNAs w/seed UAAGGCA)	− 0.965	2.41E−04
mmu-miR-10a-3p	miR-10a-3p (miRNAs w/seed AAAUUCG)	− 0.974	0.00E+00
mmu-miR-195b	miR-16-5p (and other miRNAs w/seed AGCAGCA)	− 0.991	1.70E−08
mmu-miR-33-3p	miR-33-3p (and other miRNAs w/seed AAUGUUU)	− 0.995	6.83E−05
mmu-miR-187-5p	miR-187-5p (miRNAs w/seed GGCUACA)	− 1.024	2.03E−06
mmu-miR-1a-3p	miR-1-3p (and other miRNAs w/seed GGAAUGU)	− 1.056	3.17E−06
mmu-miR-1943-5p	miR-6967-5p (and other miRNAs w/seed AGGGAGG)	− 1.074	2.77E−04
mmu-miR-1968-5p	miR-12186-3p (and other miRNAs w/seed GCAGCUG)	− 1.076	6.40E−07
mmu-miR-130a-3p	miR-130a-3p (and other miRNAs w/seed AGUGCAA)	− 1.092	0.00E+00
mmu-miR-203b-3p	miR-203b-3p (miRNAs w/seed UGAACUG)	− 1.117	8.42E−03
mmu-miR-7025-3p	miR-3590-3p (and other miRNAs w/seed AGCACAA)	− 1.17	3.46E−06
mmu-miR-133a-5p	miR-133a-5p (and other miRNAs w/seed CUGGUAA)	− 1.179	7.62E−05
mmu-miR-1941-3p	miR-1941-3p (miRNAs w/seed AUCUUAG)	− 1.256	9.72E−15
mmu-miR-1247-5p	miR-1247-5p (miRNAs w/seed CCCGUCC)	− 1.259	8.81E−03
mmu-miR-1a-1-5p	miR-1-5p (and other miRNAs w/seed CAUACUU)	− 1.337	3.66E−02
mmu-miR-383-5p	miR-383-5p (miRNAs w/seed GAUCAGA)	− 1.345	3.31E−03
mmu-miR-133a-3p	miR-133a-3p (and other miRNAs w/seed UUGGUCC)	− 1.418	8.40E−12
mmu-miR-6968-3p	miR-6968-3p (miRNAs w/seed CAAGCGC)	− 1.446	6.22E−06
mmu-miR-499-5p	miR-499-5p (and other miRNAs w/seed UAAGACU)	− 1.452	6.66E−08
mmu-miR-122-5p	miR-122-5p (miRNAs w/seed GGAGUGU)	− 1.513	3.75E−06
mmu-miR-344d-3p	miR-344d-3p (and other miRNAs w/seed AUAUAAC)	− 1.525	2.74E−09
mmu-miR-448-5p	miR-448-5p (miRNAs w/seed AACAUCC)	− 1.663	1.75E−03
mmu-miR-7010-3p	miR-597-3p (and other miRNAs w/seed GGUUCUC)	− 1.72	2.09E−02
mmu-miR-7667-5p	miR-7667-5p (miRNAs w/seed AGCCAUC)	− 1.94	4.12E−02
mmu-miR-208a-3p	miR-208a-3p (and other miRNAs w/seed UAAGACG)	− 1.95	4.32E−09
mmu-miR-208a-5p	miR-208a-5p (and other miRNAs w/seed AGCUUUU)	− 2.171	1.40E−12
mmu-miR-12181-3p	miR-12181-3p (miRNAs w/seed CCAGGCC)	− 2.595	2.40E−02
mmu-miR-1912-5p	miR-1912-5p (miRNAs w/seed GCUCAUU)	− 2.621	0.00E+00
mmu-miR-448-3p	miR-448-3p (and other miRNAs w/seed UGCAUAU)	− 2.681	0.00E+00
mmu-miR-1298-5p	miR-1298-5p (and other miRNAs w/seed UCAUUCG)	− 2.761	0.00E+00
mmu-miR-1912-3p	miR-1912-3p (miRNAs w/seed ACAGAAC)	− 2.779	2.74E−09
mmu-miR-1264-3p	miR-1264-3p (miRNAs w/seed AAAUCUU)	− 2.916	0.00E+00
mmu-miR-1298-3p	miR-1298-3p (miRNAs w/seed AUCUGGG)	− 3.143	0.00E+00
mmu-miR-1264-5p	miR-1264-5p (miRNAs w/seed GGUCCUC)	− 3.462	8.58E−03

Data show the Log2 Fold Change (log2 FC) expression of significantly disturbed microRNAs (log2 FC >  + 0.58 and < − 0.58, and FDR p-value < 0.05) between HDM and saline exposed mice, n = 14–16 per group, FDR = False Discovery Rate

### Validation of dysregulated lung miRNA signatures by QPCR

**Fig. 6:**
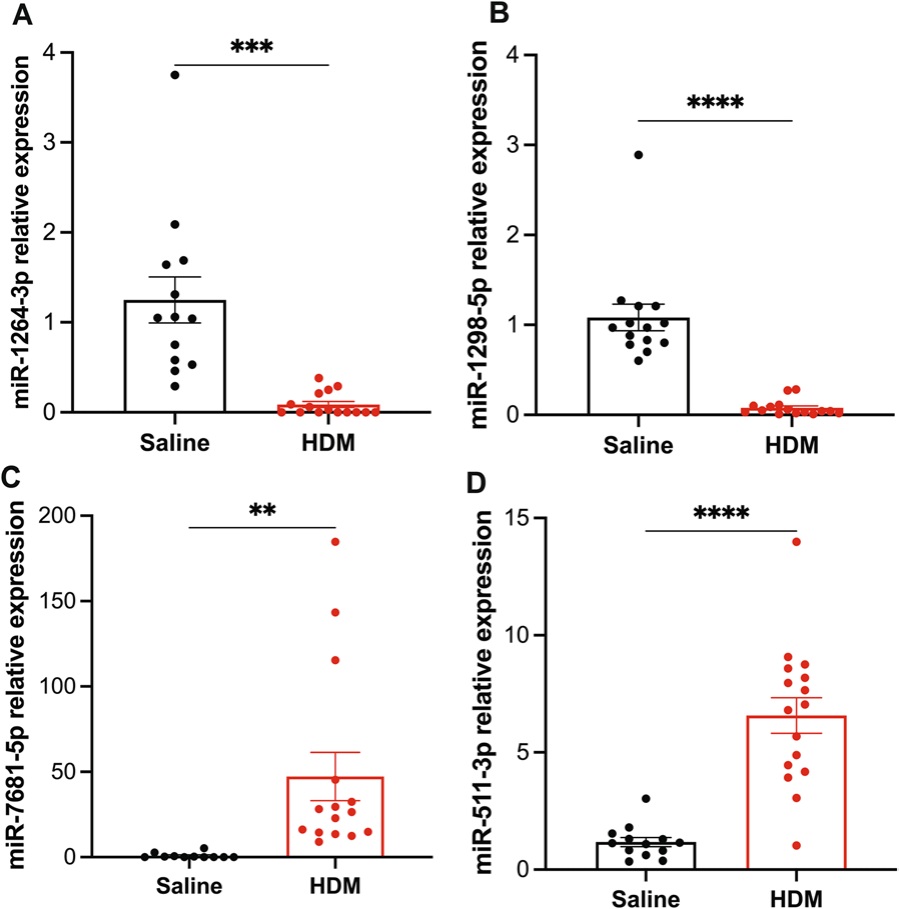
Analysis and QPCR validation of selected differentially dysregulated miRNAs. QPCR validation of downregulated (**A**, **B**) and upregulated (**C**, **D**) miRNAs. Data are expressed as mean ± SEM, n = 14–16, **p < 0.01, ***p < 0.001 and ****p < 0.0001 versus alum control, unpaired t-test with Welch's correction

The top ten downregulated miRNAs were miR-1264-5p and 3p, 1298-3p and 5p, 1912-3p and 5p, 448-3p, 12181-3p, 764-3p and 208-5p. The top ten upregulated miRNAs were miR-8090, 135b-3p and 5p, 7681-5p, 147-3p and 5p, 7062-5p, 215-3p and 5p, and 6978-3p (Table 1). Selected miRNAs (1264-3p, 1298-5p, 7681-5p, and 511-3p) among the top 25 dysregulated miRNAs were validation by QPCR. We noted that miR-1264-3p and 1298-5p are significantly decreased in HDM mice relative to control mice (Fig. 6A, B), while miR-7681-5p and miR-511-3p expression levels are substantially enhanced (Fig. 6C, D). These results are consistent with the miRNA-seq findings.

### Target identification of dysregulated lung miRNAs and pathway analysis of target genes

**Fig. 7:**
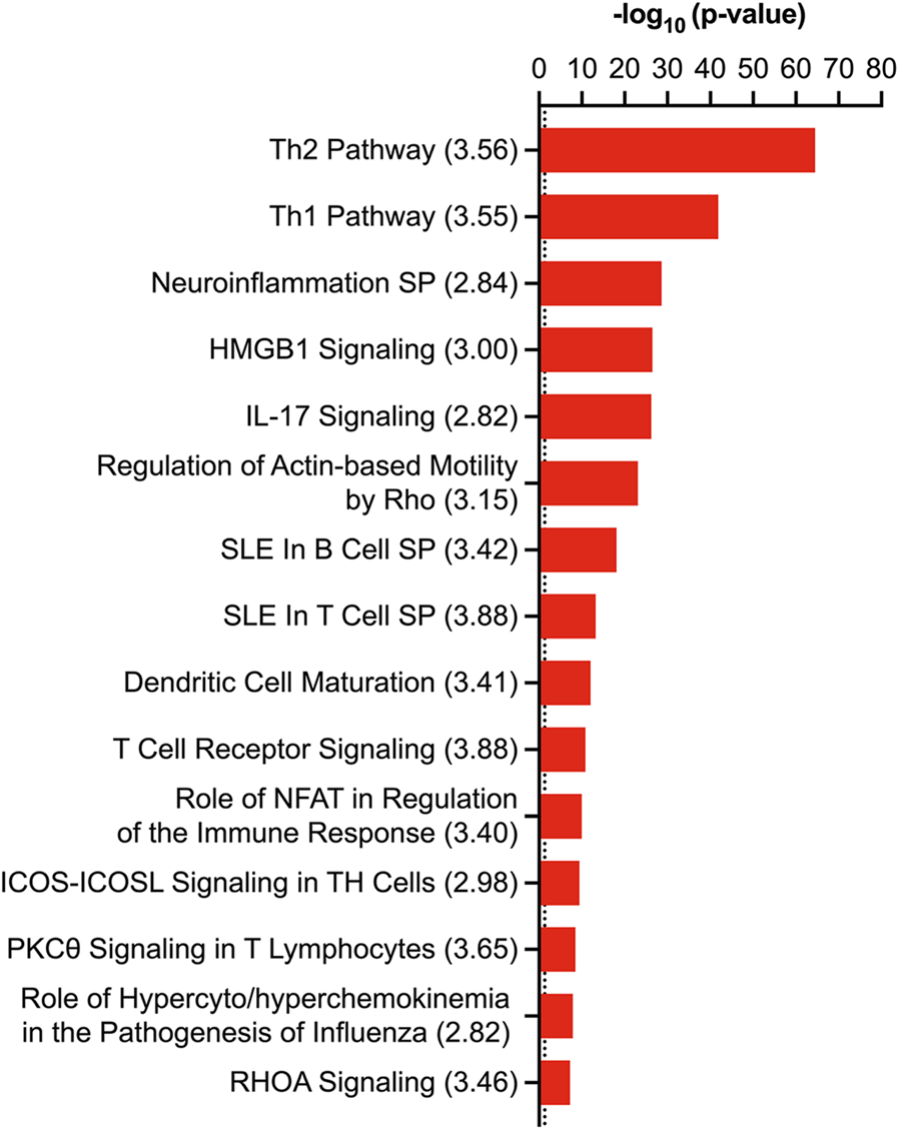
Pathway analysis. The 15-top significantly activated canonical pathways correlated with target mRNAs, with the highest z-score (> 2.8) as indicated by numbers between parentheses. All pathways shown are activated (z-score ≥  + 2). Dotted line shows the significance threshold (− log_10_ (p-value) = 1.3). Pathways with -log_10_ (p-value) > 1.3 are marked as statistically significant. SP (signaling pathway)

miRNA target analysis releveled a total of 131 microRNAs, with conserved seed sequence homology between mouse and human, targeting 211 mRNAs. There were 78 miRNAs upregulated and 53 miRNAs downregulated in HDM mice relative to saline controls. The expression pairing between miRNAs and corresponding mRNA targets is displayed in Additional file 2: Table S1. These molecules (Additional file 2: Table S1) were subject to pathway analysis to identify relevant signaling pathways and biological functions. We found that 38 canonical pathways were substantially impacted (− log_10_ (p-value) > 1.3) with significant predicted activity (Z-score ≥  + 2) or inhibition (Z-score ≤ − 2) (Additional file 1: Fig. SA). The top 15 significantly activated pathways associated with the target genes were identified using IPA and included T helper 2 and 1 (Th2 and Th1), high mobility group box protein1 (HMGB1), interleukin-17 (IL-17), T and B cell signaling, and RhoA signaling (Fig. 7).

### Functional analysis of dysregulated miRNAs and their targets

**Fig. 8:**
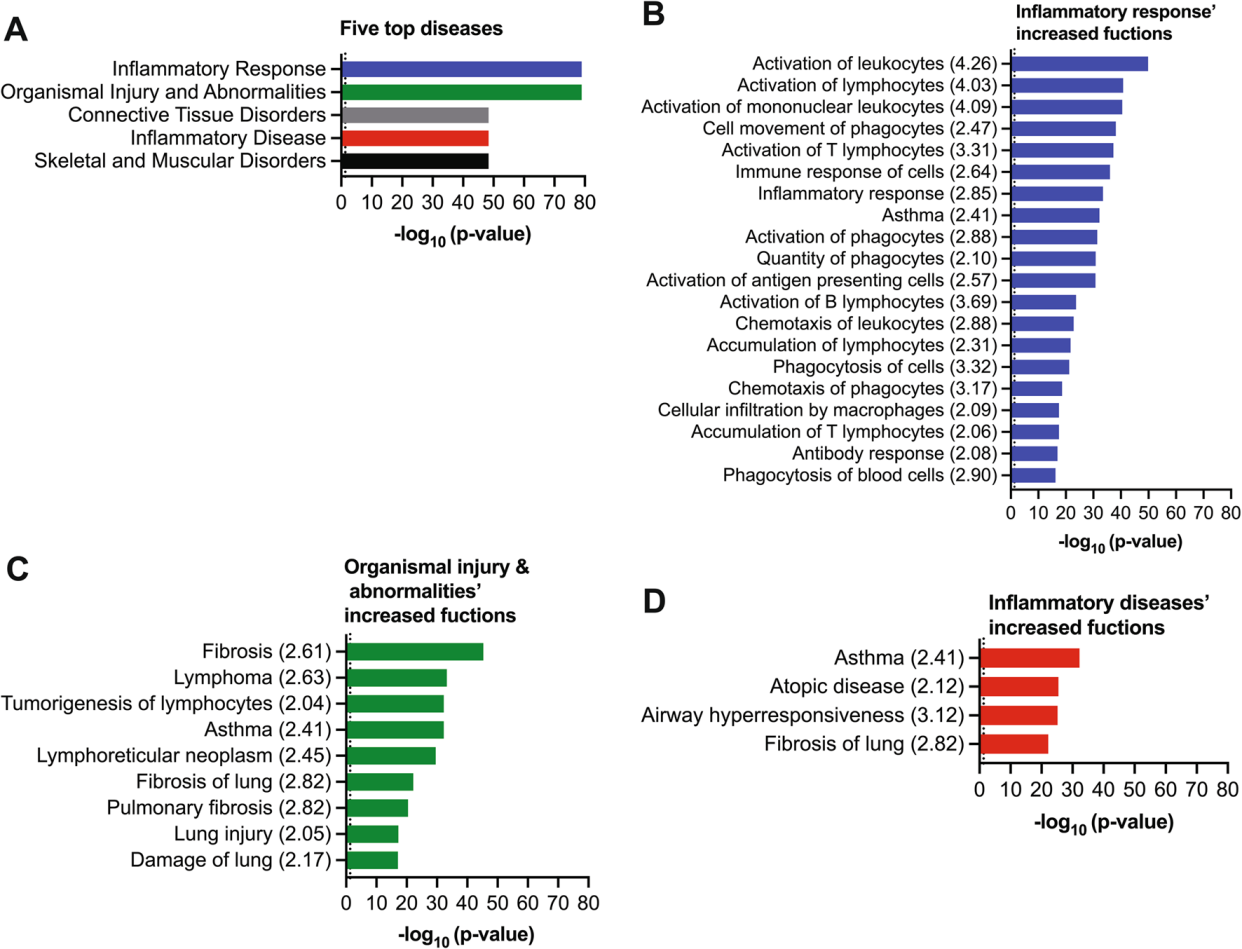
Identification of diseases and biological functions associated with miRNA signatures in the HDM mouse model. **A** Represent the five top diseases associated with the HDM model. **B**–**D** Show biological functions associated with inflammatory response (**B**), organismal injury and abnormalities (**C**), and inflammatory diseases (**D**). Z-score corresponding to each biological function is shown in the parentheses. Z-score > 2 indicate increased function. The dotted line shows the significance threshold, − log_10_ (p-value) > 1.3 are considered as statistically significant

Using pathway analysis, we identified several pathologies significantly associated with miRNAs and corresponding gene targets (Additional file 1: Fig. SB). The five top impacted pathologies were inflammation, organismal injury and abnormalities, connective tissue disorders, and skeletal and muscular disorders (Fig. 8A). The anomalies with significantly increased (z-score > 2) biological functions were inflammatory response, organismal injury and abnormalities, and inflammatory disease (Fig. 8B–D). These biological functions were mainly related to the activation, accumulation, and chemotaxis of immune cells (Fig. 8B), lung damage, injury, fibrosis (Fig. 8C), and airway hyper-responsiveness (Fig. 8D). All these functions are associated with phenotypic features of asthma. Additionally, we noted that asthma was substantially enhanced in the top-enriched disease categories (Fig. 8B–D), indicating the correlation of the lung miRNA signatures with asthma pathogenesis.

### Identification of miRNA and mRNA targets associated with an asthma network

**Fig. 9:**
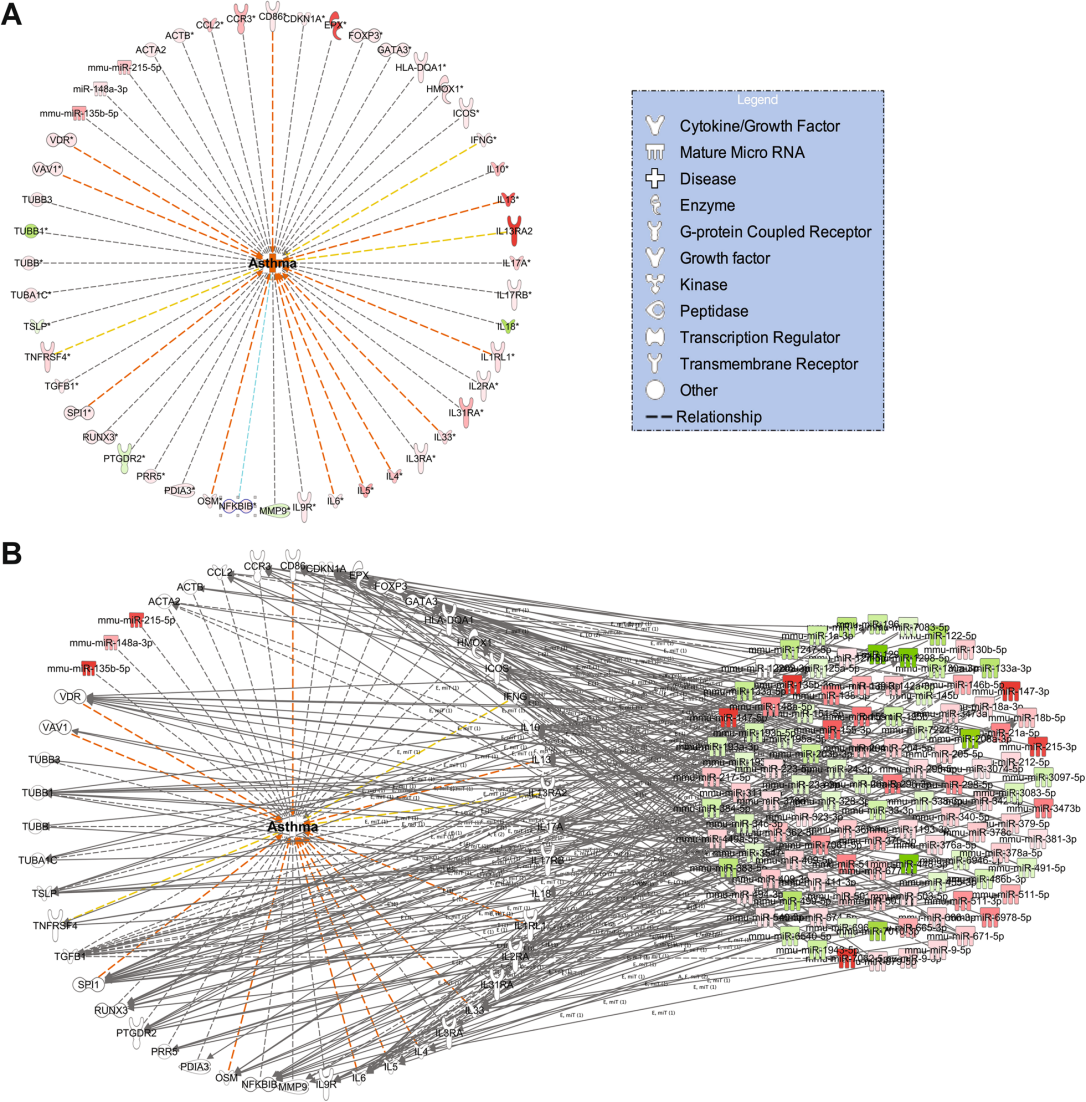
Molecules associated with an asthma network in the HDM mouse model. **A** Genes correlated with asthma pathogenesis. Nodes represent the molecules and lines indicate the relationship between two nodes. The description of the node shapes is displayed in the legends to the left side. The node color intensities show the expression level degrees between HDM mice relative to control. The downregulated molecules are in green and upregulated in pink or red. Dotted lines indicate relationships between these molecules and asthma, as predicted by the IPA findings. Red lines indicate predicted activation, yellow lines represent inconsistent findings in the literature, and grey lines mean the effect was unknown. **B** miRNA-gene network interactions. miRNAs are colored in red (upregulated) or green (downregulated), and mRNA targets are represented in the lower big circle of image 7B. Grey arrows indicate an association between miRNAs and their targets

We performed further downstream analysis, based on published data found in the IPA knowledge base, to determine genes and targeting miRNAs associated with asthma (z-score = 2.41) in mice exposed to HDM. This analysis identified 45 genes and three miRNAs (miR-135-b-5p, miR-148a-3p, and miR-215-5p). These genes included cytokines (IL-4, IL-5, and IL-13), chemokines (CCL2), growth factors (TGFβ1), peptidases (MMP9), transcription regulators (FOXP3 and GATA3), transmembrane receptors (IL13RA2 and IL17RB), enzymes and Kinases (Fig. 9A, Table 2). To connect the genes in Fig. 9A to the targeting miRNAs, we utilized the build and grow tabs in the path designer tool. These genes were targeted by 113 distinct miRNAs (Fig. 9B, Table 3). These results may indicate that most miRNAs that were differentially dysregulated in HDM mice are involved in asthma pathogenesis.

**Table 2:** Molecules directly associated with asthma

Entrez gene name	Mouse (Entrez Gene)	Log2 FC	FDR	Type(s)	Gene ID Mouse
Actin alpha 2, smooth muscle	Acta2	1.13	1.39E−19	Other	11475
Actin beta	Actb	0.82	2.13E−20	Other	11461
C–C motif chemokine ligand 2	Ccl12	3.00	1.11E−58	Cytokine	20293
C–C motif chemokine receptor 3	Ccr3	3.54	1.10E−84	G-protein coupled receptor	12771
CD86 molecule	Cd86	0.66	1.04E−09	Transmembrane receptor	12524
Cyclin dependent kinase inhibitor 1A	Cdkn1a	0.69	4.50E−05	Kinase	12575
Eosinophil peroxidase	Epx	7.02	9.26E−21	Enzyme	13861
Forkhead box P3	Foxp3	1.00	6.50E−09	Transcription regulator	20371
GATA binding protein 3	Gata3	0.74	6.19E−09	Transcription regulator	14462
Major histocompatibility complex, class II, DQ alpha 1	H2-Aa	1.38	6.01E−50	Transmembrane receptor	14960
Heme oxygenase 1	Hmox1	1.81	2.35E−39	Enzyme	15368
Inducible T cell costimulator	Icos	1.11	1.59E−09	Transmembrane receptor	54167
Interferon gamma	Ifng	0.91	2.76E−03	Cytokine	15978
Interleukin 4	Il4	2.95	8.20E−21	Cytokine	16189
Interleukin 5	Il5	4.05	9.77E−22	Cytokine	16191
Interleukin 6	Il6	1.26	3.79E−05	Cytokine	16193
Interleukin 10	Il10	3.44	1.20E−24	Cytokine	16153
Interleukin 13	Il13	6.84	2.06E−40	Cytokine	16163
Interleukin 18	Il18	− 1.86	4.28E−25	Cytokine	16,173
Interleukin 33	Il33	2.00	8.67E−87	Cytokine	77125
Interleukin 13 receptor subunit alpha 2	Il13ra2	8.17	7.85E−52	Transmembrane receptor	16165
Interleukin 17A	Il17a	2.66	5.57E−04	Cytokine	16171
Interleukin 17 receptor B	Il17rb	0.67	6.06E−04	Transmembrane receptor	50905
Interleukin 1 receptor like 1	Il1rl1	1.74	9.85E−76	Transmembrane receptor	17082
Interleukin 2 receptor subunit alpha	Il2ra	0.93	8.07E−09	Transmembrane receptor	16184
Interleukin 31 receptor A	Il31ra	3.64	2.91E−23	Transmembrane receptor	218624
Interleukin 3 receptor subunit alpha	Il3ra	0.97	7.54E−21	Transmembrane receptor	16188
Interleukin 9 receptor	Il9r	1.26	4.96E−10	Transmembrane receptor	16199
–	mmu-miR-135b-5p	4.01	0.00E+00	Mature microRNA	
–	mmu-miR-148a-3p	1.53	0.00E+00	Mature microRNA	
–	mmu-miR-215-5p	2.81	0.00E+00	Mature microRNA	
Matrix metallopeptidase 9	Mmp9	− 0.84	4.85E−05	Peptidase	17395
NFKB inhibitor beta	Nfkbib	0.63	2.26E−11	Transcription regulator	18036
oncostatin M	Osm	0.61	4.91E−04	Cytokine	18413
Protein disulfide isomerase family A member 3	Pdia3	1.03	4.25E−28	Peptidase	14827
Proline rich 5	Prr5	1.26	1.77E−18	Other	109270
Prostaglandin D2 receptor 2	Ptgdr2	− 0.85	1.44E−03	G-protein coupled receptor	14764
RUNX family transcription factor 3	Runx3	0.81	9.25E−08	Transcription regulator	12399
Spi-1 proto-oncogene	Spi1	1.14	3.33E−35	Transcription regulator	20375
Transforming growth factor beta 1	Tgfb1	0.63	2.18E−16	Growth factor	21803
TNF receptor superfamily member 4	Tnfrsf4	2.15	2.47E−28	Transmembrane receptor	22163
Thymic stromal lymphopoietin	Tslp	− 0.58	4.52E−05	Cytokine	53603
Tubulin alpha 1c	Tuba1c	1.40	2.23E−48	Other	22146
Tubulin beta class I	Tubb5	0.63	4.01E−13	Other	22154
Tubulin beta 1 class VI	Tubb1	− 1.63	3.65E−08	Other	545486
Tubulin beta 3 class III	Tubb3	1.21	2.11E−03	Other	22152
Vav guanine nucleotide exchange factor 1	Vav1	0.91	1.66E−20	Transcription regulator	22324
Vitamin D receptor	Vdr	1.40	4.89E−47	Transcription regulator	22337

Data show Log2 Fold Change (log2 FC), FDR values, IDs and cellular localization of genes associated with asthma

**Table 3:** miRNAs associated with asthma network

miRNA ID	miRNA Symbol	Log2 FC	FDR
mmu-miR-135b-3p	miR-135b-3p (and other miRNAs w/seed UGUAGGG)	4.21	0.00E+00
mmu-miR-135b-5p	miR-135a-5p (and other miRNAs w/seed AUGGCUU)	4.01	0.00E+00
mmu-miR-147-3p	miR-147 (and other miRNAs w/seed UGUGCGG)	3.96	0.00E+00
mmu-miR-7062-5p	miR-7062-5p (and other miRNAs w/seed GGAGGCC)	3.57	5.12E−06
mmu-miR-147-5p	miR-147-5p (and other miRNAs w/seed GGAAACA)	3.22	0.00E+00
mmu-miR-215-3p	miR-215-3p (miRNAs w/seed CUGUCAU)	2.96	3.75E−06
mmu-miR-215-5p	miR-192-5p (and other miRNAs w/seed UGACCUA)	2.81	0.00E+00
mmu-miR-155-3p	miR-155-3p (miRNAs w/seed UCCUACC)	2.56	2.79E−02
mmu-miR-155-5p	miR-155-5p (miRNAs w/seed UAAUGCU)	2.22	0.00E+00
mmu-miR-296-3p	miR-296-3p (miRNAs w/seed AGGGUUG)	2.17	0.00E+00
mmu-miR-3473b	miR-3473b (and other miRNAs w/seed GGCUGGA)	2.11	9.89E−11
mmu-miR-5120	miR-4260 (and other miRNAs w/seed UUGGGGC)	2.05	4.92E−02
mmu-miR-21a-5p	miR-21-5p (and other miRNAs w/seed AGCUUAU)	2.03	0.00E+00
mmu-miR-148a-5p	miR-148a-5p (and other miRNAs w/seed AAGUUCU)	1.99	0.00E+00
mmu-miR-6978-5p	miR-6782-5p (and other miRNAs w/seed AGGGGUG)	1.99	2.82E−02
mmu-miR-138-5p	miR-138-5p (miRNAs w/seed GCUGGUG)	1.98	0.00E+00
mmu-miR-298-5p	miR-298-5p (and other miRNAs w/seed GCAGAGG)	1.95	0.00E+00
mmu-miR-511-3p	miR-511-3p (miRNAs w/seed AUGUGUA)	1.85	0.00E+00
mmu-miR-7061-5p	miR-3688-3p (and other miRNAs w/seed AUGGAAA)	1.76	1.87E−03
mmu-miR-146b-5p	miR-146a-5p (and other miRNAs w/seed GAGAACU)	1.56	0.00E+00
mmu-miR-148a-3p	miR-148a-3p (and other miRNAs w/seed CAGUGCA)	1.53	0.00E+00
mmu-miR-139-3p	miR-139-3p (miRNAs w/seed GGAGACG)	1.51	1.05E−10
mmu-miR-665-3p	miR-665 (and other miRNAs w/seed CCAGGAG)	1.50	1.27E−08
mmu-miR-511-5p	miR-511-5p (miRNAs w/seed UGCCUUU)	1.48	0.00E+00
mmu-miR-370-3p	miR-370-3p (and other miRNAs w/seed CCUGCUG)	1.39	1.31E−05
mmu-miR-204-3p	miR-204-3p (and other miRNAs w/seed CUGGGAA)	1.26	4.37E−02
mmu-miR-362-5p	miR-362-5p (and other miRNAs w/seed AUCCUUG)	1.24	0.00E+00
mmu-miR-217-5p	miR-217-5p (and other miRNAs w/seed ACUGCAU)	1.20	1.13E−02
mmu-miR-18b-5p	miR-18a-5p (and other miRNAs w/seed AAGGUGC)	1.19	3.58E−07
mmu-miR-677-5p	miR-4276 (and other miRNAs w/seed UCAGUGA)	1.15	2.11E−02
mmu-miR-142a-3p	miR-142-3p (and other miRNAs w/seed GUAGUGU)	1.11	3.71E−10
mmu-miR-409-3p	miR-409-3p (miRNAs w/seed AAUGUUG)	1.06	1.25E−12
mmu-miR-369-3p	miR-369-3p (miRNAs w/seed AUAAUAC)	1.05	9.66E−08
mmu-miR-378c	miR-378a-3p (and other miRNAs w/seed CUGGACU)	1.05	0.00E+00
mmu-miR-501-5p	miR-501-5p (miRNAs w/seed AUCCUUU)	1.05	1.65E−10
mmu-miR-204-5p	miR-204-5p (and other miRNAs w/seed UCCCUUU)	1.01	7.05E−09
mmu-miR-130b-5p	miR-130b-5p (and other miRNAs w/seed CUCUUUC)	1.00	1.41E−08
mmu-miR-671-5p	miR-671-5p (miRNAs w/seed GGAAGCC)	0.98	2.49E−12
mmu-miR-3110-5p	miR-3110-5p (and other miRNAs w/seed UCUGCCU)	0.98	1.57E−02
mmu-miR-501-3p	miR-501-3p (and other miRNAs w/seed AUGCACC)	0.97	3.27E−05
mmu-miR-340-5p	miR-340-5p (miRNAs w/seed UAUAAAG)	0.95	4.50E−10
mmu-miR-205-5p	miR-205-5p (and other miRNAs w/seed CCUUCAU)	0.95	2.70E−03
mmu-miR-378a-5p	miR-378a-5p (miRNAs w/seed UCCUGAC)	0.94	0.00E+00
mmu-miR-449a-5p	miR-34a-5p (and other miRNAs w/seed GGCAGUG)	0.93	7.45E−05
mmu-miR-379-5p	miR-379-5p (and other miRNAs w/seed GGUAGAC)	0.92	6.75E−13
mmu-miR-409-5p	miR-409-5p (and other miRNAs w/seed GGUUACC)	0.91	2.73E−05
mmu-miR-666-3p	miR-666-3p (miRNAs w/seed GCUGCAG)	0.90	6.09E−03
mmu-miR-342-5p	miR-342-5p (and other miRNAs w/seed GGGGUGC)	0.89	9.09E−11
mmu-miR-3473a	miR-185-5p (and other miRNAs w/seed GGAGAGA)	0.89	2.66E−06
mmu-miR-18a-3p	miR-18a-3p (and other miRNAs w/seed CUGCCCU)	0.84	2.63E−07
mmu-miR-9-3p	miR-9-3p (and other miRNAs w/seed UAAAGCU)	0.84	1.29E−04
mmu-miR-1193-3p	miR-370-5p (and other miRNAs w/seed AGGUCAC)	0.79	1.38E−06
mmu-miR-212-5p	miR-212-5p (miRNAs w/seed CCUUGGC)	0.79	3.07E−05
mmu-miR-9-5p	miR-9-5p (and other miRNAs w/seed CUUUGGU)	0.77	5.18E−07
mmu-miR-376a-5p	miR-376a-5p (and other miRNAs w/seed GUAGAUU)	0.75	4.75E−02
mmu-miR-411-3p	miR-411-3p (and other miRNAs w/seed AUGUAAC)	0.74	6.43E−03
mmu-miR-296-5p	miR-296-5p (miRNAs w/seed GGGCCCC)	0.73	6.02E−04
mmu-miR-574-5p	miR-574-5p (miRNAs w/seed GAGUGUG)	0.72	2.72E−03
mmu-miR-223-5p	miR-223-5p (miRNAs w/seed GUGUAUU)	0.71	6.45E−05
mmu-miR-503-5p	miR-503-5p (miRNAs w/seed AGCAGCG)	0.71	1.00E−11
mmu-miR-378d	miR-3176 (and other miRNAs w/seed CUGGCCU)	0.71	9.87E−10
mmu-miR-494-3p	miR-494-3p (miRNAs w/seed GAAACAU)	0.69	4.43E−08
mmu-miR-879-5p	miR-879-5p (and other miRNAs w/seed GAGGCUU)	0.69	1.67E−02
mmu-miR-323-3p	miR-323-3p (and other miRNAs w/seed ACAUUAC)	0.66	3.40E−02
mmu-miR-540-3p	miR-540-3p (and other miRNAs w/seed GGUCAGA)	0.62	1.20E−02
mmu-miR-3074-5p	miR-3074-5p (miRNAs w/seed UUCCUGC)	0.62	2.79E−02
mmu-miR-381-3p	miR-381-3p (and other miRNAs w/seed AUACAAG)	0.61	7.24E−06
mmu-miR-127-5p	miR-127-5p (miRNAs w/seed UGAAGCU)	0.60	1.49E−05
mmu-miR-6964-3p	miR-578 (and other miRNAs w/seed UUCUUGU)	− 0.62	2.22E−02
mmu-miR-125a-5p	miR-125b-5p (and other miRNAs w/seed CCCUGAG)	− 0.63	4.76E−07
mmu-miR-193a-5p	miR-193a-5p (miRNAs w/seed GGGUCUU)	− 0.64	3.91E−04
mmu-miR-12202-3p	miR-12202-3p (and other miRNAs w/seed CUUCUCU)	− 0.64	4.31E−02
mmu-miR-24-3p	miR-24-3p (and other miRNAs w/seed GGCUCAG)	− 0.65	5.01E−15
mmu-miR-328-3p	miR-328-3p (and other miRNAs w/seed UGGCCCU)	− 0.66	7.86E−03
mmu-miR-3547-3p	miR-3547 (and other miRNAs w/seed GAGCACC)	− 0.67	2.54E−02
mmu-miR-455-3p	miR-455-3p (miRNAs w/seed CAGUCCA)	− 0.68	6.98E−06
mmu-miR-7083-5p	miR-12197-3p (and other miRNAs w/seed CGGGGCU)	− 0.68	2.56E−02
mmu-miR-338-3p	miR-338-3p (miRNAs w/seed CCAGCAU)	− 0.69	4.17E-06
mmu-miR-23a-3p	miR-23a-3p (and other miRNAs w/seed UCACAUU)	− 0.69	0.00E+00
mmu-miR-195a-3p	miR-195-3p (and other miRNAs w/seed CAAUAUU)	− 0.71	1.67E−08
mmu-miR-6946-3p	miR-4659a-3p (and other miRNAs w/seed UUCUUCU)	− 0.72	2.13E−04
mmu-miR-7224-3p	miR-181a-2-3p (and other miRNAs w/seed CCACUGA)	− 0.73	2.20E−03
mmu-miR-491-5p	miR-491-5p (and other miRNAs w/seed GUGGGGA)	− 0.73	2.16E−05
mmu-miR-151-5p	miR-151-5p (and other miRNAs w/seed CGAGGAG)	− 0.74	2.19E−10
mmu-miR-145b	miR-145-5p (and other miRNAs w/seed UCCAGUU)	− 0.74	4.11E−05
mmu-miR-26a-5p	miR-26a-5p (and other miRNAs w/seed UCAAGUA)	− 0.75	0.00E+00
mmu-miR-34c-3p	miR-34c-3p (and other miRNAs w/seed AUCACUA)	− 0.80	1.97E−06
mmu-miR-3083-5p	miR-3083-5p (and other miRNAs w/seed GGCUGGG)	− 0.83	3.78E−02
mmu-miR-193b-3p	miR-193a-3p (and other miRNAs w/seed ACUGGCC)	− 0.86	4.34E−03
mmu-miR-486b-3p	miR-486-3p (and other miRNAs w/seed GGGGCAG)	− 0.88	1.99E−02
mmu-miR-193b-5p	miR-193b-5p (miRNAs w/seed GGGGUUU)	− 0.90	2.67E−02
mmu-miR-3097-5p	miR-3097-5p (and other miRNAs w/seed ACAGGUG)	− 0.91	3.02E−03
mmu-miR-384-5p	miR-30c-5p (and other miRNAs w/seed GUAAACA)	− 0.96	3.05E−02
mmu-miR-6540-5p	miR-5624-3p (and other miRNAs w/seed UAAGGCA)	− 0.96	2.41E−04
mmu-miR-195b	miR-16-5p (and other miRNAs w/seed AGCAGCA)	− 0.99	1.70E−08
mmu-miR-33-3p	miR-33-3p (and other miRNAs w/seed AAUGUUU)	− 1.00	6.83E−05
mmu-miR-1a-3p	miR-1-3p (and other miRNAs w/seed GGAAUGU)	− 1.06	3.17E−06
mmu-miR-1943-5p	miR-6967-5p (and other miRNAs w/seed AGGGAGG)	− 1.07	2.77E−04
mmu-miR-1968-5p	miR-12186-3p (and other miRNAs w/seed GCAGCUG)	− 1.08	6.40E−07
mmu-miR-130a-3p	miR-130a-3p (and other miRNAs w/seed AGUGCAA)	− 1.09	0.00E+00
mmu-miR-203b-3p	miR-203b-3p (miRNAs w/seed UGAACUG)	− 1.12	8.42E−03
mmu-miR-133a-5p	miR-133a-5p (and other miRNAs w/seed CUGGUAA)	− 1.18	7.62E−05
mmu-miR-1247-5p	miR-1247-5p (miRNAs w/seed CCCGUCC)	− 1.26	8.81E−03
mmu-miR-1a-1-5p	miR-1-5p (and other miRNAs w/seed CAUACUU)	− 1.34	3.66E−02
mmu-miR-383-5p	miR-383-5p (miRNAs w/seed GAUCAGA)	− 1.35	3.31E−03
mmu-miR-133a-3p	miR-133a-3p (and other miRNAs w/seed UUGGUCC)	− 1.42	8.40E−12
mmu-miR-499-5p	miR-499-5p (and other miRNAs w/seed UAAGACU)	− 1.45	6.66E−08
mmu-miR-122-5p	miR-122-5p (miRNAs w/seed GGAGUGU)	− 1.51	3.75E−06
mmu-miR-7010-3p	miR-597-3p (and other miRNAs w/seed GGUUCUC)	− 1.72	2.09E−02
mmu-miR-208a-3p	miR-208a-3p (and other miRNAs w/seed UAAGACG)	− 1.95	4.32E−09
mmu-miR-448-3p	miR-448-3p (and other miRNAs w/seed UGCAUAU)	− 2.68	0.00E+00
mmu-miR-1298-5p	miR-1298-5p (and other miRNAs w/seed UCAUUCG)	− 2.76	0.00E+00
mmu-miR-1298-3p	miR-1298-3p (miRNAs w/seed AUCUGGG)	− 3.14	0.00E+00

### Identification of miRNA biomarkers for asthma

**Fig. 10:**
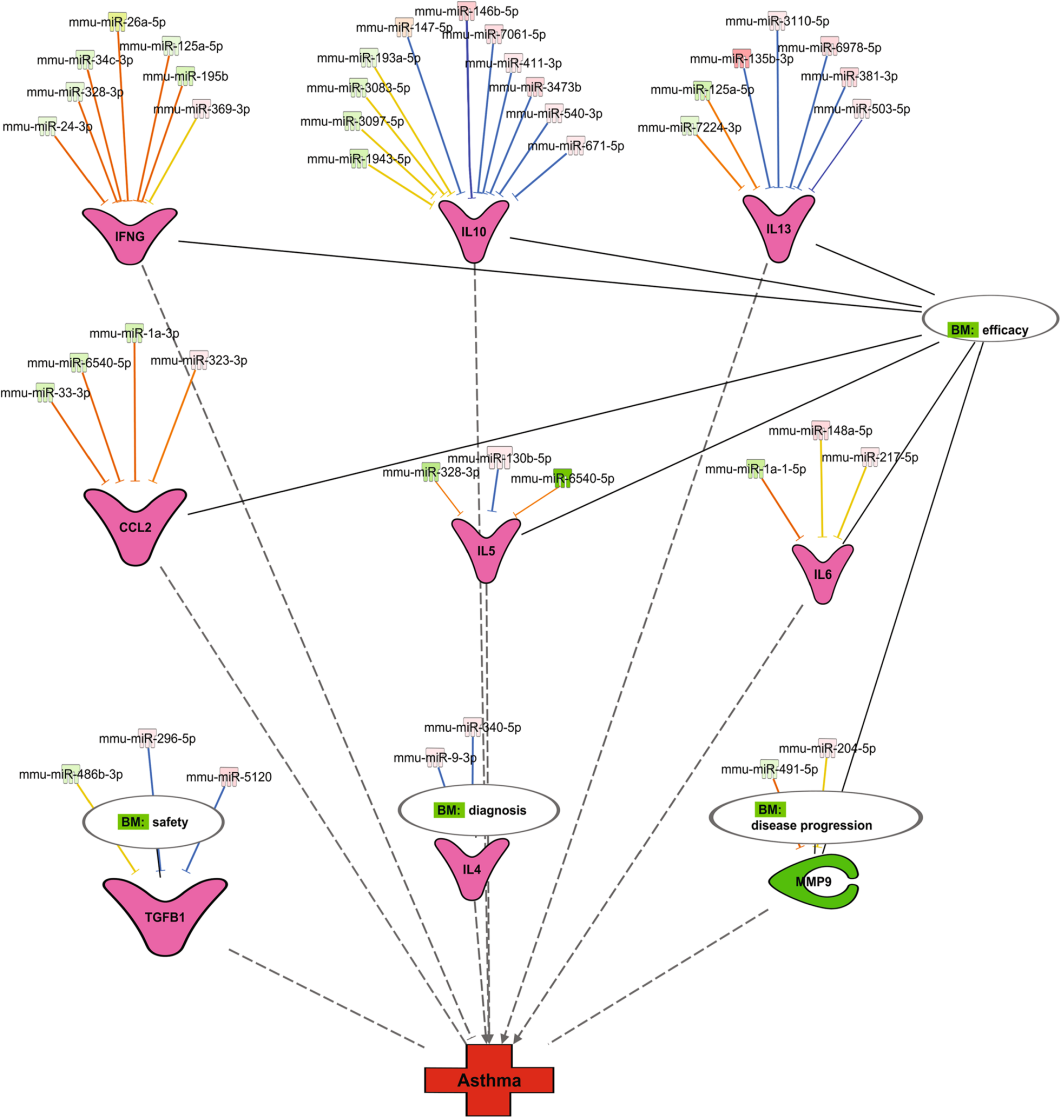
The miRNA biomarker candidates for asthma. Biomarker analysis was performed using prior knowledge from IPA. There are four miRNA biomarkers categories (efficacy, safety, diagnosis, and disease progression), and each category is connected to the molecules in the network by solid black lines. Nodes represent the molecules and lines indicate the relationship between two nodes. Green nodes represent downregulated miRNAs, and the upregulated ones are in pink or red. All mRNA targets, except MMP9, are represented in V-shaped dark pink. Each group of miRNAs is pointing to its specific mRNA target. BM: Biomarker

To investigate which miRNAs, serve as potential biomarkers, we first chose genes in Fig. 9A that were used in previous studies as biomarkers for asthma [20–24]. We found nine gene candidates (Fig. 10), including interferon gamma (IFNγ), Th2 cytokines (IL-4, IL-5, IL-6, IL-10, and IL-13), monocyte chemoattractant protein-1 (MCP-1/CCL2), transforming growth factor beta 1 (TGFβ1), and matrix metallopeptidase 9 (MMP9). These genes were grouped into four biomarker categories for asthma diagnosis, prognosis, and response of therapy (efficacy and safety) (Fig. 10). All nine genes were significantly dysregulated in our data set. The expressions of IFNγ, IL-4, IL-5, IL-6, IL-10, IL-13, MCP-1/CCL2, and TGFβ1 were increased and MMP9 was downregulated (Fig. 10, Additional file 3: Table S2). Next, we identified the targeting miRNAs for each of the genes mentioned above, and have discovered a panel of 39 miRNA candidates (Additional file 2: Table S2). The diagnostic values of candidate miRNAs were assessed using the receiver operating characteristic (ROC) analysis (Table 4). We found 17 miRNAs (miRNA-26a-5p, 24-3p, 148a-5p, 146b-5p, 147-5p, 503-5p, 135b-3p, 130b-5p, 369-3p, 195b, 3473b, 671-5p, 34c-3p, 340-5p, 204-5p, 381-3p and miR-125a-5p) with excellent predictive power (AUC: 0.92–1.00), 13 miRNAs (miRNA-9-3p, 491-5p, 33-3p, 7061-5p, 1a-3p, 6540-5p, 193a-5p, 3097-5p, 217-5p, 7224-3p, 296-5p, 411-3p and 1943-5p) with good predictive power (AUC: 0.80–0.88), and 9 miRNAs with low predictive values (AUC < 0.80) (Table 4). Overall, we have identified 30 miRNAs, with good to excellent predictive power, that may serve as potential biomarker candidates for asthma diagnosis.

**Table 4:** ROC analysis of candidate miRNA for asthma diagnosis

Receiver Operator Characteristic (ROC) analysis			
miRNA ID	AUC	95% CI	p value
mmu-miRNA-26a-5p	1.00	1.000 to 1.000	< 0.0001
mmu-miRNA-24-3p	1.00	1.000 to 1.000	< 0.0001
mmu-miRNA-148a-5p	1.00	1.000 to 1.000	< 0.0001
mmu-miRNA-146b-5p	1.00	1.000 to 1.000	< 0.0001
mmu-miRNA-147-5p	1.00	1.000 to 1.000	< 0.0001
mmu-miRNA-503-5p	1.00	1.000 to 1.000	< 0.0001
mmu-miRNA-135b-3p	1.00	0.9811 to 1.000	< 0.0001
mmu-miRNA-130b-5p	0.97	0.9149 to 1.000	< 0.0001
mmu-miRNA-369-3p	0.96	0.9035 to 1.000	< 0.0001
mmu-miRNA-195b	0.96	0.9084 to 1.000	< 0.0001
mmu-miRNA-3473b	0.96	0.9060 to 1.000	< 0.0001
mmu-miRNA-671-5p	0.96	0.8863 to 1.000	< 0.0001
mmu-miRNA-34c-3p	0.95	0.8817 to 1.000	< 0.0001
mmu-miRNA-340-5p	0.94	0.8652 to 1.000	< 0.0001
mmu-miRNA-204-5p	0.93	0.8264 to 1.000	< 0.0001
mmu-miRNA-381-3p	0.92	0.8322 to 1.000	< 0.0001
mmu-miRNA-125a-5p	0.92	0.8187 to 1.000	0.0001
mmu-miRNA-9-3p	0.88	0.7389 to 1.000	0.0004
mmu-miRNA-491-5p	0.88	0.7552 to 1.000	0.0004
mmu-miRNA-33-3p	0.84	0.6845 to 1.000	0.0014
mmu-miRNA-7061-5p	0.84	0.6846 to 0.9940	0.0016
mmu-miRNA-1a-3p	0.83	0.6864 to 0.9832	0.0018
mmu-miRNA-6540-5p	0.83	0.6672 to 1.000	0.0018
mmu-miRNA-193a-5p	0.83	0.6794 to 0.9724	0.0024
mmu-miRNA-3097-5p	0.82	0.6694 to 0.9734	0.0028
mmu-miRNA-217-5p	0.82	0.6665 to 0.9675	0.0032
mmu-miRNA-7224-3p	0.81	0.6440 to 0.9721	0.0041
mmu-miRNA-296-5p	0.81	0.6339 to 0.9821	0.0041
mmu-miRNA-411-3p	0.80	0.6441 to 0.9631	0.0047
mmu-miRNA-1943-5p	0.80	0.6331 to 0.9651	0.0053
mmu-miRNA-5120	0.78	0.6064 to 0.9471	0.01
mmu-miRNA-3110-5p	0.77	0.5996 to 0.9361	0.0126
mmu-miRNA-6978-5p	0.75	0.5762 to 0.9283	0.0188
mmu-miRNA-540-3p	0.74	0.5615 to 0.9206	0.0248
mmu-miRNA-486b-3p	0.74	0.5513 to 0.9219	0.0276
mmu-miRNA-3083-5p	0.73	0.5489 to 0.9198	0.0291
mmu-miRNA-323-3p	0.73	0.5531 to 0.9112	0.0306
mmu-miRNA-328-3p	0.69	0.4900 to 0.8939	0.0739
mmu-miRNA-1a-1-5p	0.67	0.4673 to 0.8809	0.105

AUC: area under the curve, 95% CI (confidence interval), and p-values

### Correlation of 30 miRNA biomarker candidates with asthma parameters

Next, we performed correlation analysis between the 30 miRNA biomarker candidates and various asthma parameters, including airway hyper-responsiveness (AHR), total serum IgE levels, and inflammatory cell counts. We note that 17 miRNAs showed positive correlation with asthma and 13 miRNAs displayed negative association with asthma (Table 5). Further, we note that 22 miRNAs (miRNA-135b-3p, 147-5p, 3473b, 148a-5p, 7061-5p, 146b-5p, 369-3p, 204-5p, 130b-5p, 671-5p, 340-5p, 411-3p, 503-5p, 381-3p, 125a-5p, 24-3p, 491-5p, 26a-5p, 34c-3p, 195b, 33-3p and miR-1a-3p) were significantly associated with all asthma features including IgE levels, AHR and inflammation. Four miRNAs (miRNA-9-3p, 296-5p, 193a-5p and 3097-5p) were significantly correlated with inflammation and IgE levels, but not with AHR. Two miRNAs (miR-6540-5p and 1943-5p) were inversely correlated with AHR but not associated with IgE levels or inflammation. The miR-7224-3p was negatively associated with inflammation and AHR but not linked with IgE levels, and the miRNA-217-5p was positively associated with inflammation only (Table 5). These data indicate that 22 of the 30 miRNAs tested were associated with three features of asthma, and eight of the 30 miRNAs were correlated with at least one asthma parameter.

**Table 5:** Correlation of 30 miRNA biomarker candidates with the features of asthma in HDM exposed mice

miRNAs	Airway Resistance		Total Serum IgE		Inflammatory cell counts in BAL	
miRNA ID	Spearman r	p-value	Spearman r	p-value	Spearman r	p-value
mmu-miRNA-135b-3p	0.584	0.0027	0.719	0.0002	0.814	< 0.0001
mmu-miRNA-147-5p	0.637	0.0008	0.848	1	0.779	< 0.0001
mmu-miRNA-3473b	0.529	0.0078	0.758	< 0.0001	0.662	< 0.0001
mmu-miRNA-148a-5p	0.552	0.0051	0.827	< 0.0001	0.701	< 0.0001
mmu-miRNA-7061-5p	0.436	0.0333	0.540	0.0095	0.561	0.0015
mmu-miRNA-146b-5p	0.504	0.0121	0.767	< 0.0001	0.829	< 0.0001
mmu-miRNA-217-5p	0.369	0.0756	0.392	0.0714	0.419	0.0236
mmu-miRNA-369-3p	0.508	0.0113	0.787	< 0.0001	0.614	0.0004
mmu-miRNA-204-5p	0.584	0.0027	0.732	0.0001	0.599	0.0006
mmu-miRNA-130b-5p	0.578	0.0031	0.720	0.0002	0.714	< 0.0001
mmu-miRNA-671-5p	0.434	0.0341	0.862	< 0.0001	0.627	0.0003
mmu-miRNA-340-5p	0.459	0.024	0.689	0.0004	0.641	0.0002
mmu-miRNA-9-3p	0.344	0.1003	0.792	< 0.0001	0.534	0.0029
mmu-miRNA-411-3p	0.463	0.0228	0.624	0.0019	0.376	0.0442
mmu-miRNA-296-5p	0.355	0.0889	0.607	0.0027	0.410	0.027
mmu-miRNA-503-5p	0.500	0.0128	0.900	< 0.0001	0.800	< 0.0001
mmu-miRNA-381-3p	0.416	0.0434	0.774	< 0.0001	0.610	0.0004
mmu-miRNA-125a-5p	− 0.433	0.0345	− 0.703	0.0003	− 0.638	0.0002
mmu-miRNA-193a-5p	− 0.252	0.2345	− 0.670	0.0006	− 0.525	0.0034
mmu-miRNA-24-3p	− 0.563	0.0042	− 0.682	0.0005	− 0.779	0.0001
mmu-miRNA-7224-3p	− 0.444	0.03	− 0.236	0.2915	− 0.540	0.0025
mmu-miRNA-491-5p	− 0.456	0.0252	− 0.658	0.0009	− 0.506	0.0051
mmu-miRNA-26a-5p	− 0.653	0.0005	− 0.618	0.0022	− 0.772	< 0.0001
mmu-miRNA-34c-3p	− 0.635	0.0009	− 0.475	0.0255	− 0.621	0.0003
mmu-miRNA-3097-5p	− 0.385	0.063	− 0.453	0.0341	− 0.472	0.0097
mmu-miRNA-6540-5p	− 0.512	0.0105	− 0.356	0.1036	− 0.366	0.0509
mmu-miRNA-195b	− 0.637	0.0008	− 0.689	0.0004	− 0.736	< 0.0001
mmu-miRNA-33-3p	− 0.503	0.0123	− 0.482	0.0232	− 0.422	0.0227
mmu-miRNA-1a-3p	− 0.471	0.0201	− 0.441	0.0399	− 0.585	0.0009
mmu-miRNA-1943-5p	− 0.518	0.0095	− 0.156	0.487	− 0.258	0.1772

The correlation coefficient, r, was calculated using Spearman's test, p < 0.05 was considered significant. BAL: bronchoalveolar lavage

## Discussion

The primary goal of this study was to identify miRNA biomarkers for asthma using a house dust mite (HDM) mouse model of allergic inflammation. Although several common allergens are used to induce allergic inflammation, HDM remains the most clinically-relevant antigen, as it affects nearly 85% of asthma patients worldwide [25]. Even though HDM is a well-established model, the outcomes of the allergic response and associated molecular signatures, such as miRNA profiles, can vary significantly depending on the dose of the allergen, sensitization route, mouse strain, and protocol used. Our results show that HDM- exposed mice exhibited an increased airway resistance, prominent airway inflammation and thickening, elevated serum HDM-specific IgE, and increased production of key inflammatory mediators. These results are in line with previous studies and indicate that the HDM protocol used in this study elicited a potent allergic inflammatory response with airway remodeling that recapitulates key features of human asthma [17, 26].

We utilized miRNA sequencing followed by bioinformatic analysis to identify miRNA signatures in lung tissue and serum of HDM-exposed mice relative to the saline control group. The comparison between lung tissue and serum miRNA profiles revealed 213 miRNAs significantly dysregulated in lung tissue, with the log2 fold change expression cut off (> + 0.58 and cut off < − 0.58) and FDR p-values < 0.05. Unexpectedly, only miR-146b-5p was substantially upregulated in the serum. These results suggest that lung tissue is a more reliable source of miRNA-based biomarker discovery for asthma than circulating miRNAs. To the best of our knowledge, this is the second study to analyze the free circulating miRNA profiles in a murine model of allergic inflammation. The first group (Milger K. et al.) to profile plasma in a mouse model of inflammation identified 11 significantly dysregulated miRNAs with the fold change (FC) from 0.59 to 1.75 and p < 0.01 [16]. While we only found one miRNA significantly upregulated (FC = 2.5 and FDR p-value = 5.13E−13). In Milger K. et al.’s study, miRNA signatures analysis was done using a focus panel containing 179 known miRNAs in plasma samples from female mice exposed to HDM or PBS. However, our study used serum from male and female mice, and miRNA profiles were determined via deep sequencing. The discrepancy between Miller et al.’s results and ours may be due to the gender and experimental methodology differences between the two studies.

The lung is a complex organ comprised of various cell types, including epithelial, smooth muscle, fibroblast, and endothelial cells [27] that undergoes structural changes and inflammatory cell recruitment when exposed to allergens such as HDM. Thus, analysis of miRNA signatures from whole lungs likely reflects a pool of various miRNAs from specific lung cell types and infiltrating immune cells, such as eosinophils. This would probably be more beneficial in unveiling the accurate landscape of the miRNA signature profile for asthma rather than using a specific lung cell type. Based on this rationale, we decided to focus solely on lung miRNA signatures since miR-146b-5p was the only miRNA dysregulated in serum, and miR146b-5p was also included in the whole lung analysis.

Target analysis of lung miRNA signatures showed 131 microRNAs targeting 211 mRNAs. These miRNAs had conserved seed sequence homology with human miRNAs, indicating their relevance to human asthma. Pathway analysis demonstrated that the mRNA targets were implicated in various immune and inflammatory signaling pathways, and Th2 signaling was the most significantly enriched pathway. Functional analysis indicated that the dysregulated miRNAs and corresponding mRNA targets were involved in several inflammatory responses and diseases, specifically in immune cell activation, accumulation, and chemotaxis, lung damage, lung injury, lung fibrosis, airway hyper-responsiveness, and asthma. Interestingly, all these biological functions are involved in the phenotypic features of asthma. Taken together, these findings suggest that lung miRNA signatures identified herein are associated with Th2-mediated asthma pathogenesis, the most predominant feature observed in mice models of allergic inflammation, including the BALB/c mouse strain [28]. Therefore, these miRNA signatures would be specifically relevant for the diagnosis of patients with the Th2-high asthma phenotype but not those with other asthmatic phenotypes, such as Th2-low asthma.

The asthma network analysis unveiled 113 miRNAs targeting 45 genes. These genes included cytokines (IL-4, IL-5, and IL-13), chemokines (CCL2), growth factors (TGFβ1), peptidases (MMP9), transcription regulators (FOXP3 and GATA3), transmembrane receptors (IL13RA2 and IL17RB), enzymes and kinases that were significantly dysregulated with 41 genes were upregulated and four were downregulated in our dataset. These results confirm the implication of these genes in asthma and agree with previous human asthma studies [3, 29].

The miRNA-gene network analysis identified 39 miRNAs out of 113 miRNAs as potential biomarkers for asthma. ROC analysis showed that 30 of the 39 candidates had a substantial predictive power (AUC ≥ 80 for each miRNA) for asthma diagnosis. The association of the 30 miRNA candidates with asthma was performed via correlation analysis between these miRNAs and three asthma parameters, including airway hyperresponsiveness (AHR), total serum IgE levels, and BAL inflammatory cell counts. The correlation analysis indicated that 17 of the 30 miRNAs were positively correlated with asthma, and 13 of the 30 miRNAs were inversely linked to asthma. Further, 22 of the 30 miRNAs were associated with IgE levels, AHR, and inflammation and eight were correlated with at least one asthma parameters. The association of most of these miRNAs with several asthma features indicate that they may serve as viable candidates for asthma diagnosis.

Out of the 30 identified miRNAs, 18 (miR-135b-3p, 147-5p, 148a-5p, 146b-5p, 204-5p, 130b-5p, 671-5p, 340-5p, 9-3p, 296-5p, 503-5p, 125a-5p, 193a-5p, 24-3p, 26a-5p, 34c-3p, 195b, and miR-1a-3p) had been previously linked to asthma pathogenesis [12, 30–33]. For instance, miRNA-296-5p, 125a-5p, 195b, and 26a-5p were correlated with lung function [15, 34, 35]. The miRNA-296-5p expression was associated with AHR in response to increasing methacholine PC_20_ in the childhood asthma management program cohort [34]. The miR-26a-5p expression was associated with FEV_1_/FVC ratio in asthma patients [15]. The miR-34c-3p was downregulated in asthmatic patients and its overexpression inhibited airway remodeling via the insulin growth factor binding protein-3 (IGFBP-3) signaling [36]. Other miRNAs such as miRNA-671-5p, miR-135b-3p, 147-5p, 340-5p and miR-1a-3p were dysregulated in murine models of asthma [8, 37]. These findings agree with our results and confirm the implication of several of these miRNAs in human asthma. Surprisingly, among the 30 identified potential biomarkers, the miR-146b-5p was the only upregulated miRNA in common between serum and lung samples. This finding corroborates several studies that also found an increased miR-146b-5p expression in various murine models of allergic inflammation, as well as in asthma patients [8, 10, 11]. Our findings confirm previous studies and indicate that miRNA-146b-5p is a viable biomarker for asthma.

This study also identified 12 novel miRNAs (miR-3473b, 7061-5p, 217-5p, 369-3p, 411-3p, 381-3p, miR-7224-3p, 491-5p, 3097-5p, miR-6540-5p, 33-3p, and 1943-5p) implicated in asthma pathogenesis. Interestingly, some of these miRNAs, specifically miR-3473b, 217-5p, and 369-3p, display a role in human inflammatory diseases other than the lung. For instance, the miR-3473b has been shown to participate in post-stroke neuroinflammation injury by targeting the suppressor of cytokine signaling 3 (SOCS3), a crucial negative regulator of Th2-mediated allergic responses [38]. Additionally, the miR-3473b targets the triggering receptor expressed on myeloid cells 2 (TREM2) and Unc-51 like autophagy activating kinase 1 (ULK1) to regulate the inflammatory response, such as the secretion of TNF-α and IL-1β, in Parkinson's disease [39]. Next, the downregulation of miR-217 was associated with decreased endothelial cell proliferation and enhanced secretion levels of IL-6, IL-1β, ICAM-1, and TNF-α. Whereas its overexpression was parallel with a reverse phenomenon [40]. Further, the upregulation of miR-369-3p in dendritic cells was associated with a decrease in pro-inflammatory (TNFα, IL-6, IL-12, IL-1α, IL-1β) and an increase in anti-inflammatory (IL-10 and IL-1RA) cytokine secretions in response to lipopolysaccharides (LPS)-induced inflammatory response, suggesting a possible anti-inflammatory role of this miRNA [41]. Overall, these findings indicate that miRNAs 3473b, 217-5p, and 369-3p participate in allergic inflammation by potentially regulating the production of various inflammatory cytokines.

In addition to the inflammatory role of some miRNAs, other candidates have been involved in fibrosis or proliferation. For instance, miR-411-3p has been shown to alleviate pulmonary and skin fibrosis through modulation of TGF-β/Smad ubiquitin regulatory factor 2 (Smurf 2) signaling, whereas miR-491-5p inhibits the non-small cell lung cancer (NSCLC) cell proliferation and migration by targeting forkhead box P4 (FOXP4), a transcription factor involved in the regulation of various cancers [42–44]. However, the implication of the remaining seven novel miRNAs (miR-7061-5p, 381-3p, miR-7224-3p, 3097-5p, miR-6540-5p, 33-3p, and 1943-5p) in lung biology still unknown.

Similar studies have been done in ovalbumin models using either male or female mice [8, 37, 45]. However, our study has several strengths, including using the HDM mouse model, a most clinically-relevant model of allergic inflammation. Additionally, we surveyed an extensive number of miRNAs using male and female mice. Further, each of the 30 miRNA candidates had good predictive power (AUC ≥ 80%), making these miRNAs stand-alone biomarkers for asthma diagnosis. The weakness of the study is that we did not confirm our findings in human samples. Despite this, several miRNAs discovered herein were also implicated in human asthma, indicating the validity of HDM models in profiling lung miRNAs. Subsequent studies will be performed to analyze how these miRNAs regulate asthma. Further, we will test if these miRNAs can serve as biomarkers in clinical settings using lung biopsy samples from asthmatic and healthy individuals.

## Conclusions/future directions

These results suggest that lung tissue is a more reliable source of miRNA-based biomarker discovery for asthma than circulating miRNAs. Overall, these findings indicate that mouse models of inflammation might not be ideal models for circulating miRNA studies. Regardless of this plausible specific pitfall, the HDM model used herein was very useful in recapacitating all key features of human asthma, leading to the discovery of unique lung miRNA signatures for allergic asthma. Although most of these miRNAs were previously implicated with asthma, we have identified 12 novel biomarkers. Subsequent studies will validate the efficiency of these miRNA in asthmatic patients.

## Supplementary Information

**Additional file 1.** (SA) Canonical pathways significantly dysregulated in response to HDM exposure. DR (differential regulation) and CP (cytokine production). (SB) Functional analysis of dysregulated miRNAs and their mRNA targets, all significantly impacted diseases (z-score > 2). Red bars mean activation (z-score ≥ + 2) and blue bars indicate inhibition (z-score ≤ − 2). Dotted line shows the significance threshold (− log_10_ (p-value) = 1.3), and − log_10_ (p-value) > 1.3 is considered as statistically significant.

**Additional file 2: Table S1.** Expression pairing between dysregulated microRNAs and mRNA targets.

**Additional file 3:** **Table S2.** Potential miRNA biomarkers for asthma.

## Data Availability

The data discussed in this manuscript were deposited in the NCBI’s Gene Expression Omnibus (https://www.ncbi.nlm.nih.gov/geo) and are accessible through GEO Series accession number GSE214937.

## Abbreviations

miR: MicroRNA
HDM: House dust mite
AHR: Hyper-responsiveness
BAL: Bronchoalveolar lavage
IPA: Ingenuity pathway analysis
TGF-β: Transforming growth factor beta
IgE: Immunoglobulin E
ROC: Receiver operating characteristic
AUC: Area under the curve
Smurf2: Smad ubiquitin regulatory factor 2
FOXP4: Forkhead box P4
