# Supplementary figures and images for: Gene network analysis for identification of microRNA biomarkers for asthma
Source: Respir Res. 2022 Dec 26;23:378. doi: 10.1186/s12931-022-02304-2 (PMC9793650; doi:10.1186/s12931-022-02304-2)

Figure S

A

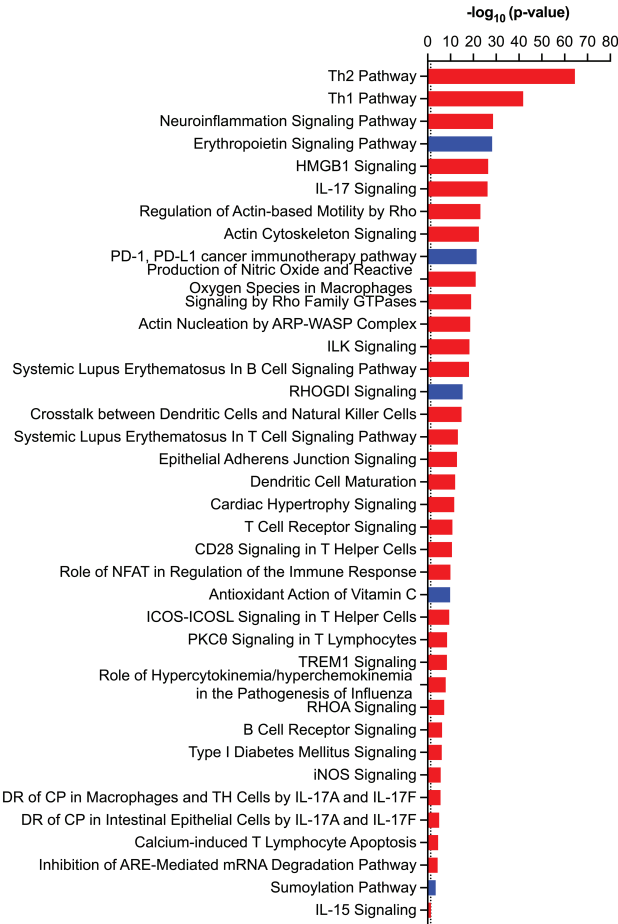

B

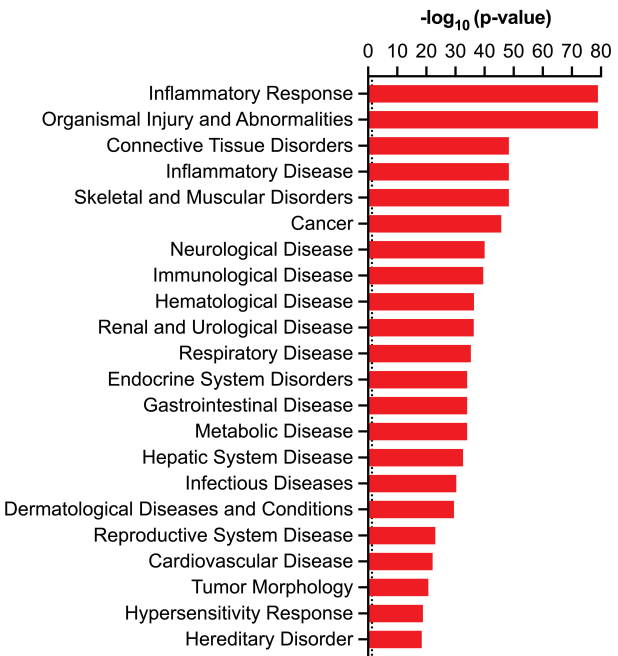

Supplement: Supplementary file 1 — Additional file 1. (SA) Canonical pathways significantly dysregulated in response to HDM exposure. DR (differential regulation) and CP (cytokine production). (SB) Functional analysis of dysregulated miRNAs and their mRNA targets, all significantly impacted diseases (z-score > 2). Red bars mean activation (z-score ≥ + 2) and blue bars indicate inhibition (z-score ≤ − 2). Dotted line shows the significance threshold (− log10 (p-value) = 1.3), and − log10 (p-value) > 1.3 is considered as statistically significant. [file 12931_2022_2304_MOESM1_ESM.pdf]
