# Supplementary material for: Gene network analysis for identification of microRNA biomarkers for asthma
Source: Respir Res. 2022 Dec 26;23:378. doi: 10.1186/s12931-022-02304-2 (PMC9793650; doi:10.1186/s12931-022-02304-2)
Supplement: Supplementary file 3 — Additional file 3: Table S2. Potential miRNA biomarkers for asthma. [file 12931_2022_2304_MOESM3_ESM.docx]

| \| **Genes** \| \| \| \| **Targeting miRNAs** \| \| \| \| \| \| --- \| --- \| --- \| --- \| --- \| --- \| --- \| --- \| --- \| \| **Symbol** \| **Mouse**  **Gene ID** \| **Log2 FC** \| **FDR** \| **miRNA ID** \| **Log2 FC** \| **FDR** \| **Confidence** \| **Source** \| \| CCL2 \| 20293 \| 2.99 \| 1.11E-58 \| mmu-miRNA-323-3p \| 0.66 \| 3.40E-02 \| Moderate \| TagetScan \| \| mmu-miRNA-1a-3p \| -1.05 \| 3.17E-06 \| High \| TagetScan \| \| mmu-miRNA-6540-5p \| -0.96 \| 2.41E-04 \| High \| TagetScan \| \| mmu-miRNA-33-3p \| -0.99 \| 6.83E-05 \| High \| TagetScan \| \| IFNG \| 15978 \| 0.91 \| 2.76E-03 \| mmu-miRNA-369-3p \| 1.05 \| 9.66E-08 \| Moderate \| TagetScan \| \| mmu-miRNA-195b \| -0.99 \| 1.70E-08 \| Exp. Obser. \| [1] \| \| mmu-miRNA-125a-5p \| -0.63 \| 4.76E-07 \| Moderate \| TagetScan \| \| mmu-miRNA-26a-5p \| -0.75 \| 0.00E+00 \| Moderate \| TagetScan \| \| mmu-miRNA-34c-3p \| -0.79 \| 1.97E-06 \| Moderate \| TagetScan \| \| mmu-miRNA-328-3p \| -0.65 \| 7.86E-03 \| Moderate \| TagetScan \| \| mmu-miRNA-24-3p \| -0.64 \| 5.01E-15 \| High \| TagetScan \| \| IL4 \| 16189 \| 2.95 \| 8.20E-21 \| mmu-miRNA-340-5p \| 0.95 \| 4.50E-10 \| Moderate \| TagetScan \| \| mmu-miRNA-9-3p \| 0.84 \| 1.29E-04 \| High \| TagetScan \| \| IL5 \| 16191 \| 4.05 \| 9.77E-22 \| mmu-miRNA-130b-5p \| 1.00 \| 1.41E-08 \| Moderate \| TagetScan \| \| mmu-miRNA-328-3p \| -0.65 \| 7.86E-03 \| High \| TagetScan \| \| mmu-miRNA-6540-5p \| -0.96 \| 2.41E-04 \| Moderate \| TagetScan \| \| IL6 \| 16193 \| 1.26 \| 3.79E-05 \| mmu-miRNA-217-5p \| 1.20 \| 1.13E-02 \| Moderate \| TagetScan \| \| mmu-miRNA-148a-5p \| 1.99 \| 0.00E+00 \| Moderate \| TagetScan \| \| mmu-miRNA-1a-1-5p \| -1.33 \| 3.66E-02 \| High \| TagetScan \| \| IL10 \| 16153 \| 3.44 \| 1.20E-24 \| mmu-miRNA-671-5p \| 0.98 \| 2.49E-12 \| Moderate \| TagetScan \| \| mmu-miRNA-540-3p \| 0.61 \| 1.20E-02 \| Moderate \| TagetScan \| \| mmu-miRNA-3473b \| 2.10 \| 9.89E-11 \| Moderate \| TagetScan \| \| mmu-miRNA-411-3p \| 0.74 \| 6.43E-03 \| High \| TagetScan \| \| mmu-miRNA-7061-5p \| 1.76 \| 1.87E-03 \| Moderate \| TagetScan \| \| mmu-miRNA-146b-5p \| 1.56 \| 0.00E+00 \| Exp. Obser. \| [2] \| \| mmu-miRNA-147-5p \| 3.22 \| 0.00E+00 \| Moderate \| TagetScan \| \| mmu-miRNA-193a-5p \| -0.63 \| 3.91E-04 \| Exp. Obser. \| [3] \| \| mmu-miRNA-3083-5p \| -0.83 \| 3.78E-02 \| Moderate \| TagetScan \| \| mmu-miRNA-3097-5p \| -0.90 \| 3.02E-03 \| High \| TagetScan \| \| mmu-miRNA-1943-5p \| -1.07 \| 2.77E-04 \| Moderate \| TagetScan \| \| IL13 \| 16163 \| 6.84 \| 2.06E-40 \| mmu-miRNA-503-5p \| 0.70 \| 1.00E-11 \| Moderate \| TagetScan \| \| mmu-miRNA-381-3p \| 0.61 \| 7.24E-06 \| Moderate \| TagetScan \| \| mmu-miRNA-6978-5p \| 1.99 \| 2.82E-02 \| Moderate \| TagetScan \| \| mmu-miRNA-3110-5p \| 0.98 \| 1.57E-02 \| Moderate \| TagetScan \| \| mmu-miRNA-135b-3p \| 4.20 \| 0.00E+00 \| Moderate \| TagetScan \| \| mmu-miRNA-125a-5p \| -0.63 \| 4.76E-07 \| Moderate \| TagetScan \| \| mmu-miRNA-7224-3p \| -0.72 \| 2.20E-03 \| Moderate \| TagetScan \| \| MMP9 \| 17395 \| -0.84 \| 4.85E-05 \| mmu-miRNA-204-5p \| 1.01 \| 7.05E-09 \| Exp. Obser. \| miRecords \| \| mmu-miRNA-491-5p \| -0.73 \| 2.16E-05 \| Moderate \| TagetScan \| \| TGFB1 \| 21803 \| 0.63 \| 2.18E-16 \| mmu-miRNA-5120 \| 2.05 \| 4.92E-02 \| Moderate \| TagetScan \| \| mmu-miRNA-296-5p \| 0.73 \| 6.02E-04 \| High \| TagetScan \| \| mmu-miRNA-486b-3p \| -0.88 \| 1.99E-02 \| Moderate \| TagetScan \| |
| --- | --- | --- | --- | --- | --- | --- | --- | --- | --- | --- | --- | --- | --- | --- | --- | --- | --- | --- | --- | --- | --- | --- | --- | --- | --- | --- | --- | --- | --- | --- | --- | --- | --- | --- | --- | --- | --- | --- | --- | --- | --- | --- | --- | --- | --- | --- | --- | --- | --- | --- | --- | --- | --- | --- | --- | --- | --- | --- | --- | --- | --- | --- | --- | --- | --- | --- | --- | --- | --- | --- | --- | --- | --- | --- | --- | --- | --- | --- | --- | --- | --- | --- | --- | --- | --- | --- | --- | --- | --- | --- | --- | --- | --- | --- | --- | --- | --- | --- | --- | --- | --- | --- | --- | --- | --- | --- | --- | --- | --- | --- | --- | --- | --- | --- | --- | --- | --- | --- | --- | --- | --- | --- | --- | --- | --- | --- | --- | --- | --- | --- | --- | --- | --- | --- | --- | --- | --- | --- | --- | --- | --- | --- | --- | --- | --- | --- | --- | --- | --- | --- | --- | --- | --- | --- | --- | --- | --- | --- | --- | --- | --- | --- | --- | --- | --- | --- | --- | --- | --- | --- | --- | --- | --- | --- | --- | --- | --- | --- | --- | --- | --- | --- | --- | --- | --- | --- | --- | --- | --- | --- | --- | --- | --- | --- | --- | --- | --- | --- | --- | --- | --- | --- | --- | --- | --- | --- | --- | --- | --- | --- | --- | --- | --- | --- | --- | --- | --- | --- | --- | --- | --- | --- | --- | --- | --- | --- | --- | --- | --- | --- | --- | --- | --- | --- | --- | --- | --- | --- | --- | --- | --- | --- | --- | --- | --- | --- | --- | --- | --- | --- | --- | --- | --- | --- | --- | --- | --- | --- | --- | --- | --- | --- | --- | --- |

**Table S2. Potential miRNA biomarkers for asthma**. Data show Log2 Fold Change (log2 FC), FDR values, IDs, targeting confidence and source. Experimentally observed (Exp. Obser.).

1. Sullivan RP, Leong JW, Schneider SE, Keppel CR, Germino E, French AR, et al. MicroRNA-deficient NK cells exhibit decreased survival but enhanced function. J Immunol 2012; 188:3019-30.

2. Recchiuti A, Krishnamoorthy S, Fredman G, Chiang N, Serhan CN. MicroRNAs in resolution of acute inflammation: identification of novel resolvin D1-miRNA circuits. Faseb j 2011; 25:544-60.

3. Tufekci KU, Oner MG, Genc S, Genc K. MicroRNAs and Multiple Sclerosis. Autoimmune Dis 2010; 2011:807426.
